# Xanthohumol inhibits PRRSV proliferation and alleviates oxidative stress induced by PRRSV via the Nrf2–HMOX1 axis

**DOI:** 10.1186/s13567-019-0679-2

**Published:** 2019-09-11

**Authors:** Xuewei Liu, Zhongbao Song, Juan Bai, Hans Nauwynck, Yongxiang Zhao, Ping Jiang

**Affiliations:** 1 Key Laboratory of Animal Disease Diagnostics and Immunology, Ministry of Agriculture, MOE International Joint Collaborative Research Laboratory for Animal Health & Food Safety, College of Veterinary Medicine, Nanjing Agricultural University, Nanjing, 210095 China; 2 Laboratory of Virology, Faculty of Veterinary Medicine, Ghent University, Salisburylaan 133, 9820 Merelbeke, Belgium; 3 Jiangsu Co-innovation Center for Prevention and Control of Important Animal Infectious Diseases and Zoonoses, Yangzhou, China

## Abstract

Porcine reproductive and respiratory syndrome virus (PRRSV) is a prevalent and endemic swine pathogen that causes significant economic losses in the global swine industry. Commercial vaccines provide limited protection against this virus, and no highly effective therapeutic drugs are yet available. In this study, we first screened a library of 386 natural products and found that xanthohumol (Xn), a prenylated flavonoid found in hops, displayed high anti-PRRSV activity by inhibiting PRRSV adsorption onto and internalization into cells. Transcriptome sequencing revealed that Xn treatment stimulates genes associated with the antioxidant response in the nuclear factor-erythroid 2-related factor 2 (Nrf2) signalling pathway. Xn causes increased expression of Nrf2, HMOX1, GCLC, GCLM, and NQO1 in Marc-145 cells. The action of Xn against PRRSV proliferation depends on Nrf2 in Marc-145 cells and porcine alveolar macrophages (PAMs). This finding suggests that Xn significantly inhibits PRRSV proliferation and decreases viral-induced oxidative stress by activating the Nrf2–HMOX1 pathway. This information should be helpful for developing a novel prophylactic and therapeutic strategy against PRRSV infection.

## Introduction

Porcine reproductive and respiratory syndrome (PRRS) is one of the most economically detrimental swine diseases worldwide. Infection is characterized by reproductive failure and preterm birth in sows as well as dyspnoea of piglets and fattening pigs [1, 2]. The aetiologic agent is PRRS virus, a positive-sense, single-stranded RNA virus belonging to the family *Arteriviridae* in the order *Nidovirales*. PRRSV is divided into two genotypes, the European genotype (type I) and the North American genotype (type II), which share approximately 60% identity in amino acid sequences [3]. The mutation rate of PRRSV is 3.29 × 10^−3^ substitutions per nucleotide site per year in China alone [4, 5]. Commercially available vaccines can provide only incomplete protection. Modified live virus (MLV) vaccines protect against homologous strains [6], but they have the potential risk of reverting to a more virulent form or recombining with pandemic strains to form a novel genotype strain during serial passage in pigs [7]. These problems highlight the need for alternative approaches to control this disease. A combination of pharmacological intervention and vaccination is worthy of further inquiry.

The interaction between PRRSV and pigs has been well studied. Several host antiviral factors, such as the interferon-stimulated genes (ISGs) viperin [8], myxovirus resistance 2 (Mx2) [9], 2′,5′-oligoadenylate synthetase 1 (OAS1) [10], interferon-induced protein with tetratricopeptide repeats 3 (IFIT3) [11], and CH25H [12, 13], have been reported to have antiviral activity against PRRSV infection. Some microRNAs, small interfering RNAs (siRNAs) [14], and short-hairpin RNAs (shRNAs) [15] have also been shown to inhibit PRRSV replication. Meanwhile, some natural compounds and compositions have also been reported to have anti-PRRSV activity in vitro, such as sodium tanshinone IIA sulfonate [16], proanthocyanidin A2 [17], Griffithsin [18], and (-)-epigallocatechin-3-gallate [19]. However, the mechanism of antiviral activity is not deeply understood. In this study, we screened a library of 386 natural products and found that xanthohumol, a prenylated flavonoid extracted from the hop plant *Humulus lupulus* L, significantly inhibited the early stages of PRRSV infection and inhibited virally induced oxidative stress by activating the Nrf2–HMOX1 pathway in Marc-145 cells and PAMs, demonstrating excellent potential as a therapeutic agent.

## Materials and methods

### Cells, viruses, and reagents

Marc-145 cells (an African green embryonic kidney epithelial cell line, ATCC) were cultured in Dulbecco’s modified Eagle’s medium (Invitrogen, USA) supplemented with 10% foetal bovine serum (FBS; GIBCO) at 37 °C in a humidified atmosphere containing 5% CO_2_. Porcine alveolar macrophages (PAMs) were collected from lung lavages of 6-week-old Yorkshire pigs (free of PRRSV, PCV2, PRV), as previously described, and cultured in RPMI-1640 (GIBCO) containing 10% FBS at 37 °C. Three North American genotype 2 PRRSV strains were employed. The highly pathogenic PRRSV strain BB0907 (GenBank accession no. HQ315835.1), which is maintained in our laboratory, was used for all experiments and is represented by “PRRSV” in this article. The PRRSV strains S1 (GenBank accession no. DQ459471.1) and FJ1402 (GenBank accession no. KX169191.1) were also used but are specifically mentioned by name (S1, a classical strain; FJ1402, a NADC30-like strain).

Xanthohumol, purity > 99%, was used for in vitro experiments (Selleck Chemicals, Houston TX, USA). Protoporphyrin IX cobalt chloride (CoPP), an inducer of *HMOX1* expression, was purchased from Sigma (St. Louis, MO, USA).

### Screening of a natural product library

**Figure 1:**
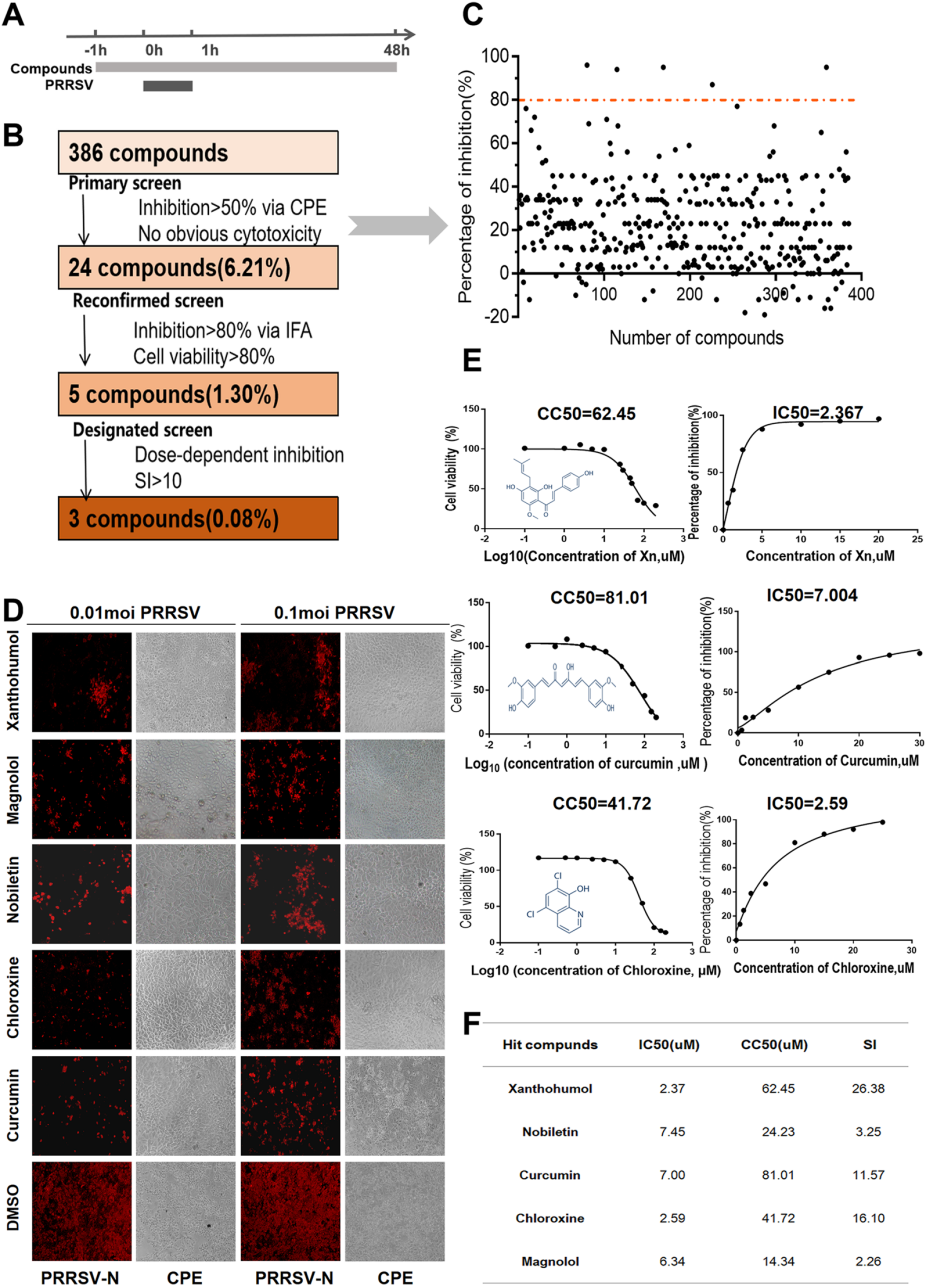
**Screening protocol for PRRSV inhibitors. A** Screening procedure time course. Marc-145 cells were treated with 10 µM compound for 1 h and then infected with PRRSV (0.01 MOI) for 1 h. Cells were washed with PBS and then incubated in medium containing 10 µM compound for another 48 h. **B** Screening process flowchart. The criteria for passing the primary screen were that the compound had no apparent cytotoxicity and that it reduced CPE by at least 50% compared with that of the positive controls. The criteria for passing the secondary screen were that the compound had to leave cells at least 80% viable and had to inhibit PRRSV (0.01 MOI and 0.1 MOI) by more than 80%. Compounds that passed the tertiary screen inhibited PRRSV in a dose-dependent manner and had a selective index greater than ten. **C** Each dot represents the percent inhibition of PRRSV (0.01 MOI) achieved with each compound. The dots located above the dotted line indicate 80% or greater inhibition. **D** IFA of infected cells treated with one of the five designated compounds. PRRSV N-protein is coloured red, and brightfield-imaged cells show CPE. **E** IC_50_ and CC_50_ curves of the 5 designated compounds. The structure of each compound is inset. **F** SIs of the 5 designated compounds. Selectivity index (SI) = CC_50_/IC_50_

A library of 386 natural products was purchased from Selleck Chemicals (Houston, TX, USA). These compounds were stored as 10 mM stock solutions in DMSO at 80 °C until use. The workflow for screening the library is diagrammed in Figures 1A and B. Marc-145 cells were seeded in 96-well plates at 2 × 10^4^ cells per well. When approximately 60% confluent, the cells were treated with 10 µM compound or DMSO (1 µL) for 1 h and then infected with PRRSV (0.01 MOI) or mock infected for 1 h. Cells were then washed with PBS, and culture medium containing 10 µM compound was added back to each well. At 48 h post-infection (hpi), the percentage of inhibition was calculated by CPE and IFA. For each assay, there were two technical replicates, i.e., two compound-infected groups, two DMSO-PRRSV infected groups, and two DMSO-mock infected groups.

During the primary screening, compounds were screened out if they resulted in any observable cytotoxicity or demonstrated less than a 50% reduction of CPE compared with that of the DMSO control group. For the second round of screening, cell viability had to be 80% or greater, and the inhibition of PRRSV had to be 80% or greater (0.01 MOI and 0.1 MOI) via IFA. The 50% inhibitory concentration (IC_50_) and 50% cytotoxic concentration (CC_50_) of each remaining candidate compound were determined, and those that displayed dose-dependent inhibition of PRRSV and had a selectivity index greater than 10 were considered for further study.

### Cell viability assay

Concentrations of Xn were added to cultures of Marc-145 cells and incubated for 48 h at 37 °C. Cell viability was tested using a Cell Counting Kit-8 (CCK-8) (Beyotime, Nanjing, China) following the manufacturer’s instructions. The 50% cytotoxic concentration (CC_50_) was calculated using GraphPad Prism 7.0 software. DMSO was used as the negative control.

### PRRSV infectivity inhibition assay

An indirect immunofluorescence assay (IFA) was used to examine the effect of Xn on PRRSV-infected Marc-145 cells. Two-fold serially diluted compounds were added to the culture medium of Marc-145 cells (final concentrations were 1 µM to 20 µM). DMSO was used as the negative control. PRRSV (0.01 MOI) was then added onto the cells, and cultures were incubated for 48 h at 37 °C. Cells were then fixed with 4% paraformaldehyde in PBS and permeabilized with 0.1% Triton X-100. After three washes with PBS, cells were incubated with an anti-PRRSV N-protein mAb (made in our laboratory) [20] for 1 h at 37 °C. Cells were washed three times with PBS and then incubated with Alexa Fluor 594-conjugated goat anti-mouse IgG (H–L) (1:200, Proteintech) for 1 h at 37 °C in the dark. Nuclei were stained with DAPI (Invitrogen, China) for 10 min at room temperature. Immunofluorescence was observed using a Nikon A1 confocal microscope (Nikon A1; Nikon, Japan). The level of fluorescence was determined using ImageJ software. GraphPad Prism 5.0 software was used to estimate the 50% inhibition concentration (IC_50_) of each compound. The selectivity index (SI) was determined by the ratio of CC_50_ to IC_50_.

### Western blot assay

Cells were lysed on ice for 15 min in lysis buffer (Beyotime, China), then resolved by SDS-PAGE and transferred to a nitrocellulose membrane. The membrane was blocked with 5% low-fat milk for 2 h at room temperature and then probed with antibodies: anti-PRRSV N-protein (1:1000), anti-β-actin (1:1000; Santa Cruz, CA, USA), anti-HMOX1 (1:1000; Proteintech, USA), and anti-human Nrf2 (1:1000; Proteintech) for 2 h at room temperature. Membranes were incubated with HRP-conjugated goat anti-mouse and anti-rabbit IgG (H–L) secondary antibodies (1:1000; Beyotime, China). Bound proteins were visualized with a Tanon 5200 chemiluminescence imaging system (Tanon, China).

### RNA extraction and quantitative real-time PCR

Total RNA was extracted from cells using a Total RNA Kit I (Omega Bio-tek). RNA was then reverse transcribed using a HiScript II 1st Strand cDNA Synthesis Kit (Vazyme, China) following the manufacturer’s instructions. Quantitative RT-PCR was performed using AceQ^®^ qPCR SYBR^®^ Green Master Mix (Vazyme, China). Data are presented as the fold change in gene expression normalized to GAPDH and relative to the mock-infected control. Each reaction was performed in triplicate, and the data are calculated as the mean (M) ± SEM. Primer sequences for genes are shown in Table 1.

**Table 1:** **Primer sequences of probes used for qPCR analysis**

Primer	Sequence (5′ → 3′)
mGAPDH-Fwd	5′ CCTTCCGTGTCCCTACTGCCAA 3′
mGAPDH-Rev	5′ GACGCCTGCTTCACCACCTTCT 3′
mHMOX-1-Fwd	5′ CTTCAAGCTGGTGATGGC 3′
mHMOX-1-Rev	5′ TGGAGCCGCTTCACATAG 3′
mNrf2-Fwd	5′ ATTCAATGATTCTGACTCTG 3′
mNrf2-Rev	5′ CGTATCCCCAGAAGAATGTA 3′
mNQO1-Fwd	5′ CATGTACTCTCTGCAAGGGA 3′
mNQO1-Rev	5′ TCCCAAATATTCTCCAGGCG 3′
mTXNRD1-Fwd	5′ TGTTGTGGGCTTTCACGTAC 3′
mTXNRD1-Rev	5′ ATGTTGTGAATACCTCTGCA 3′
mTNXIP-Fwd	5′ CGACCCTGAAAAGGTGTAC 3′
mTNXIP-Rev	5′ CGAACTTGTACTCATATTTG 3′
mGCLM-Fwd	5′ TCAGTGGGCACAGGTAAAA 3′
mGCLM-Rev	5′ TTGTTTAGCAAATGCAGTCA 3′
mGCLC-Fwd	5′ ACATGCGAAAACGGCGGAA 3′
mGCLC-Rev	5′ CGAGGGTGCTTGTTTATTGC 3′
pHMOX-1-Fwd	5′ GGCTGAGAATGCCGAGTT 3′
pHMOX-1-Rev	5′ ATGTAGCGGGTGTAGGCG 3′
PRRSV-ORF7-Fwd	5′ AAACCAGTCCAGAGGCAAG 3′
PRRSV-ORF7-Rev	5′ TCAGTCGCAAGAGGGAAAT 3′

p means pig and m means monkey

### Virus titration

Marc-145 cells grown in 96-well plates were infected with ten-fold serial dilutions of PRRSV. After 1 h at 37 °C, the culture medium was replaced with fresh DMEM-2% FBS. Viral titres were determined using endpoint dilution analysis at 5 days post-inoculation (dpi). The Reed–Muench method was used to calculate the 50% tissue culture infected dose (TCID_50_).

### Time-of-addition experiment

To evaluate which stage of the PRRSV life cycle is affected by Xn, a time-of-addition experiment was performed as shown in the timeline schematic (Figure 3A). Marc-145 cells seeded into 24-well plates were either pre-treated, co-treated, or post-treated with Xn relative to PRRSV inoculation. The experiment began when the cells reached 60% confluence and was noted as −1 h. At 0 h, culture supernatants of the pre-treated cells were replaced by DMEM-2% FBS, cells were then inoculated with PRRSV (0.01 MOI), the co-treated group was treated with 10 µM Xn and PRRSV, and the post-treated group was inoculated with PRRSV. At +1 h, the culture supernatants in the co- and post-treated groups were replaced with DMEM/2% FBS, and the post-treated group was treated with 10 µM Xn. Incubation of all groups continued for 48 h.

### Virucidal activity assay

A virucidal activity assay was performed to determine whether Xn interacts with PRRSV directly. Xn (10 µM) or DMSO was incubated with PRRSV (0.001, 0.01, and 0.1 MOI) at 37 °C for 2 h. Marc-145 cells in six-well plates were pre-cooled at 4 °C for 1 h. The culture supernatants were replaced with a mixture of Xn (10 µM) or DMSO and PRRSV. After incubation at 4 °C for another 1 h, cells were overlaid with DMEM containing 1% low-melting-point agarose (Sigma-Aldrich) and 2% FBS and incubated for 72 h at 37 °C. The cells were overlaid with 1% crystal violet in methanol and incubated for an additional 2 h at 37 °C. Plaque-forming units were counted.

### Virus adsorption assay

Marc-145 cells cultured in 24-well plates were pre-chilled at 4 °C for 1 h. Culture supernatants were then replaced by a 4 °C mixture of Xn (5, 10, and 15 µM) or DMSO and PRRSV (1 MOI) and incubated at 4 °C for another 1 h. Cells were washed with ice-cold PBS, and then the mRNA levels of PRRSV ORF7 in cells were measured using qRT-PCR.

### Virus internalization assay

Marc-145 cells were plated, grown to approximately 70% confluence, pre-treated with cycloheximide (CHX) (10 μg/mL) for 12 h, washed and incubated with PRRSV (1 MOI) for 1 h at 4 °C to allow virus attachment. Cells were washed 3 times with ice-cold PBS to remove unbound virus. Then, the culture medium was replaced with fresh DMEM containing 10 µM Xn or DMSO, and the cells were incubated for 2 h at 37 °C. Cells were washed with citrate buffer (pH 3) to remove non-internalized virus. The levels of PRRSV ORF7 mRNA were detected by qRT-PCR, and the virions in cells were detected and visualized using IFA confocal laser-scanning microscopy.

### Virus replication assay

Marc-145 cells were incubated with PRRSV (1 MOI) at 37 °C. At 6 hpi, cells were washed 3 times with PBS to remove free virus, the culture medium was replaced with fresh DMEM-2% FBS containing Xn (10 µM) or DMSO, and the cultures were incubated at 37 °C. At 7, 8, 9, and 10 hpi, PRRSV genome levels in cells were measured using qRT-PCR.

### Virus release assay

Marc-145 cells were infected with PRRSV (0.1 MOI) for 1 h at 37 °C, and the culture medium was then replaced with fresh DMEM-2% FBS. At 24 hpi, the cells were washed 3 times with PBS, the culture medium was replaced with fresh DMEM-2% FBS containing Xn (10 µM) or DMSO, and the cultures were incubated at 37 °C for 10, 30, and 60 min, at which point the supernatants were harvested. Released virus was quantified by plaque assay.

### RNA isolation, sequencing, and functional analysis

Marc-145 cells exposed to 10 μM Xn or DMSO for 4, 8, and 12 h were collected for transcriptomic analysis; for each time point, there were two biological replicates. RNA isolation and sequencing were performed by Novogene Bioinformatics Technology Co., Ltd. (Beijing, China). Sequencing libraries were generated using an NEBNext^®^ UltraTM RNA Library Prep Kit for Illumina^®^ (NEB, USA) following the manufacturer’s recommendations. Index codes were added to attribute sequences to each sample. Briefly, total RNA was isolated using TRIzol reagent (Invitrogen, NJ, USA), and genomic DNA was then removed using DNase I (Invitrogen). mRNA was purified from total RNA using poly-T oligo-attached magnetic beads. First strand cDNA was synthesized using random hexamer primers. Second strand cDNA synthesis was subsequently performed using DNA polymerase I and RNase H. The double-stranded cDNAs were purified using AMPure XP beads, then used for end reparation and “A” base addition, and finally ligated with sequencing adapters. The adaptor-ligated fragments were size selected using AMPure XP beads. After quantification using a Qubit 2.0 fluorometer (Life Technologies), cDNAs were used for PCR amplification and sequenced as 2 × 120 bp paired-end reads on an Illumina HiSeq™ 2000 sequencer (Illumina, San Diego, CA, USA). FeatureCounts v1.5.0-p3 was used to count the read numbers mapped to each gene, and then the FPKM (number of Fragments Per Kilobase of transcript sequence per Millions base pairs sequenced) of each gene was calculated based on the length of the gene. Differential expression analysis of two conditions/groups (two biological replicates per condition) was performed using the DESeq2 R package. The resulting *P*-values were adjusted using Benjamini and Hochberg’s approach for controlling the false discovery rate. Genes with an adjusted *P* value < 0.05 found by DESeq2 were assigned as differentially expressed. Finally, GO and KEGG analyses were performed to understand the effect of Xn on cell biological processes, molecular function, and cellular components. All raw sequence reads data were deposited in the NCBI Sequence Read Archive (SRA) with accession number PRJNA516508.

### Plasmid construction

Total RNA was extracted from PAMs using a Total RNA Kit I (Omega Bio-tek, Norcross, GA, USA), and cDNA synthesis was performed with SuperScript III Reverse Transcriptase (Invitrogen). HMOX1 was generated by PCR amplification of cDNA from PAMs with the oligonucleotide pair SacI HMOX1 5′-ACG AGC TCA TGG AGC GTC TGC AAC CCG ACA-3′ and NheI HMOX1 5′-CGG CTA GCT CAA GCA ACG TCC GGA ACG TCG TAC GGG TAC ATG GCA TAA AGC-3′. The sequence of the amplification product was compared to that in the NCBI database for verification (GenBank accession no. NM_001004027.1), restriction digested, and cloned into the pCAGGS vector with an HA tag at its 3′ end to produce pCAGGS-HMOX1.

### Plasmid transfection and virus challenge

To determine the effects of HMOX1 on PRRSV replication, Marc-145 cells plated in 24-well plates were transfected with 0, 0.4, 0.6 or 1.0 μg of pCAGGS-HMOX1 using Lipofectamine 3000 (Invitrogen) according to the manufacturer’s recommendations. Twenty-four hours after transfection, the cells were infected with PRRSV (0.01 MOI) and then harvested for Western blotting at 48 hpi.

### Small interfering RNA assays

A total of 2 × 10^6^ Marc-145 cells were transfected into 24-well plates with 50 pmol of siNC (negative control) and siNrf2 (GenePharma, Shanghai, China) using 5 µL of Lipofectamine RNAiMAX (Invitrogen, Carlsbad, CA, USA). After 24 h, the cells were treated with Xn and infected with PRRSV (0.01 MOI) for 36 h. Cells were harvested for Western blotting and qRT-PCR. The sequences of siRNAs targeting Nrf2 were: (a) 5′-AGA CAA ACA TTC AAG CCG-3′; (b) 5′-AGA ATA AAG TGG CTG CTC-3′.

### Reactive oxygen species (ROS) levels

Cellular levels of reactive oxygen species were determined using a dichlorofluorescein (DCF) ROS assay kit according to the manufacturer’s instructions (Beyotime Biotechnology, China). Marc-145 and PAM cells were infected with PRRSV (0.1 MOI) for 12 h and then treated with 5 µM Xn or DMSO for another 12 h. 10 mM DCF-DA was added to the cells for 30 min at 37 °C. The Marc-145 cells were washed and imaged using an inverted fluorescence microscope (Nikon). The PAMs were collected by centrifugation, washed, and suspended in cold PBS; fluorescent intensity was analysed on a FACSCalibur (BD Biosciences, San Jose, CA, USA), and the data were analysed using FlowJo version 7.6.1. Cells treated with 12 µM H_2_O_2_ for 3 h, washed, and then treated with DMSO or 5 µM Xn for another 12 h served as positive controls.

### MDA, GSH and SOD levels

Levels of superoxide dismutase (SOD), glutathione peroxidase (GSH), and malondialdehyde (MDA) in cells were determined using a Superoxide Dismutase (SOD) assay kit, a Glutathione Peroxidase (GSH-PX) assay kit, and a Microscale Malondialdehyde (MDA) assay kit, respectively, following the manufacturers’ instructions (Jiancheng Bioengineering Institute, Nanjing, China). The levels were normalized to the protein concentration determined by a bicinchoninic acid (BCA) protein assay kit (Beyotime).

### Statistical analyses

All statistical analyses were performed using GraphPad Prism (version 7.0, GraphPad Software, San Diego, CA, USA), and the data are expressed as the mean ± standard error of the mean. The significance of differences among groups was determined by one-way or two-way analysis of variance (ANOVA). Differences with *p*-values < 0.05 were considered significant and are designated with * in the figures.

## Results

### Library screening

Marc-145 cells were treated with 10 µM natural product and infected with PRRSV as detailed in the timeline (Figure 1A). The results revealed that 24 (6.21%) compounds showed no apparent cytotoxicity and reduced CPE by 50% compared to that with DMSO alone. These compounds were subjected to a second round of screening (Figure 1B). Five of these compounds produced minimal cytotoxicity and inhibited PRRSV infection (0.01 MOI and 0.1 MOI) by more than 80%, as determined by IFA (Figures 1C and D). Of these 5 compounds, only xanthohumol (Xn), curcumin, and chloroxin inhibited PRRSV in a dose-dependent manner and had a selectivity index (SI) greater than 10 (Figures 1E and F).

**Figure 2:**
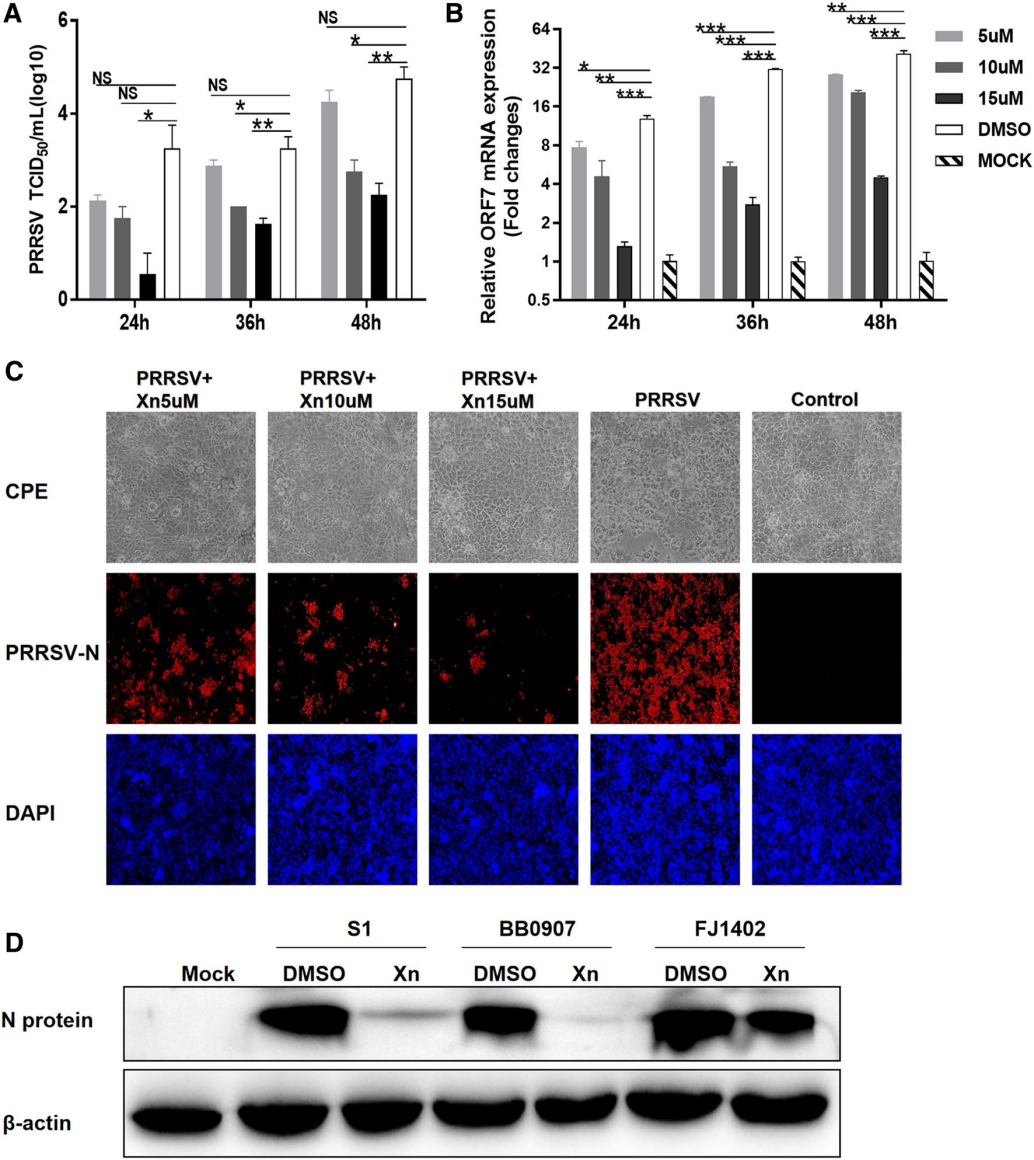
**Identification of anti-PRRSV activity of Xn in Marc-145 cells.** Marc-145 cells were pre-treated with the indicated concentrations of Xn (**A**–**C**) or 10 µM Xn (**D**) for 1 h and then infected with PRRSV (0.01 MOI) for 1 h at 37 °C. Cells were washed with PBS and then incubated in fresh medium containing Xn for 48 h (**C**) or the indicated times (**A**, **B**). DMSO served as the treatment control. **A** Culture supernatants were collected at the indicated time points for viral titration. The results are expressed as a 50% tissue culture infective dose (TCID_50_). Titres from three independent experiments are shown as the means ± SEMs (error bars). **B** Relative PRRSV ORF7 mRNA levels determined by qRT-PCR and expressed relative to ORF7 mRNA levels in mock cells. GAPDH was the internal loading control. **C** At 48 hpi, light microscopy and IFA images of Marc-145 PRRSV-infected and Xn-treated cells. Viral N-protein is red, and nuclei are blue. **D** At 48 hpi, Western blot of N-protein in cells infected with different PRRSV genotypes and treated with 10 µM Xn or DMSO. All assays were performed in triplicate, with three technical repeats for each sample. Bars represent means ± SEMs from three independent experiments. ***P < 0.001; **P < 0.01; *P < 0.05

To determine the dose range of Xn anti-PRRSV activity, Marc-145 cells were treated with 5, 10, and 15 µM Xn for 1 h and then infected with PRRSV (0.01 MOI). TCID_50_ and qRT-PCR assays performed at 24, 36, and 48 hpi showed that virus titres and ORF7 mRNA levels decreased in a dose-dependent manner at each post-infection time point (Figures 2A and B). At 48 hpi, CPE and IFA revealed that the number of infected cells in the Xn-treated groups was obviously less than that in the negative control (Figure 2C). Western blot analysis demonstrated that Xn was as effective at reducing replication of the C-PRRSV S1 strain as it was at reducing replication of the BB0907 strain and was somewhat effective at reducing replication of the NADC30-like FJ1402 strain (Figure 2D).

### Xanthohumol inhibits PRRSV infection

**Figure 3:**
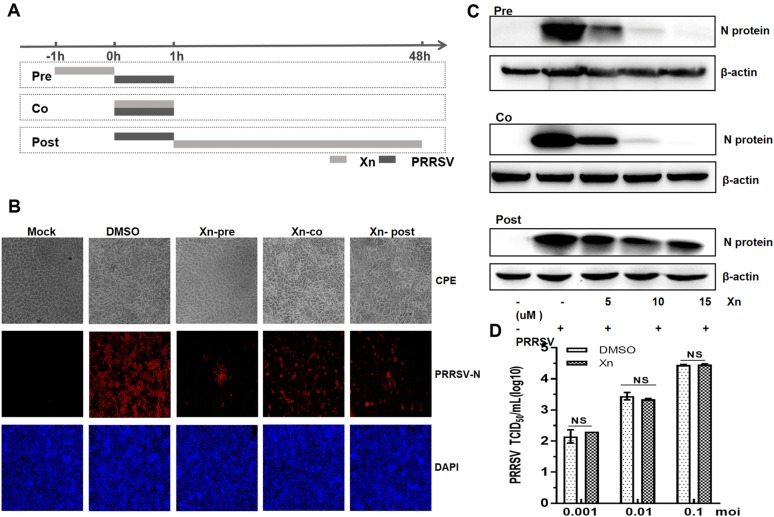
**Xanthohumol inhibits PRRSV early in infection in Marc-145 cells. A** Time-of-addition schematic. Marc-145 cells were infected with PRRSV (0.01 MOI) for 1 h (0 to 1 h), and cells were treated with 10 µM Xn at different times of infection, designated pre-treatment (pre), co-treatment (co), or post-treatment (post). **B** The effect of Xn was determined by CPE and IFA. **C** The effect of different concentrations of Xn was detected by Western blotting. **D** Xn (10 µM) or DMSO was incubated with PRRSV (0.001, 0.01, and 0.1 MOI) for 2 h at 37 °C and then inoculated into Marc-145 cells. After incubation for 48 h at 37 °C, the viral titres were detected by TCID_50_ assays. The results are representative of three independent experiments performed in triplicate

To begin to determine the mechanism of Xn inhibition of PRRSV, Marc-145 cells were treated with Xn before, during, and after PRRSV infection (Figure 3A). At 48 hpi, cells were observed for CPE, stained for IFA, and harvested for Western blotting. PRRSV inhibition was greatest in cells treated with Xn for 1 h before infection and was significantly suppressed in cells treated with Xn during infection. In cells treated with Xn for 24 h after infection, PRRSV was still suppressed but not to the extent seen in the pre- and co-treated cells (Figures 3B and C). To determine if Xn affected PRRSV directly, 10 µM Xn was mixed with virus, incubated for 2 h at 37 °C and then added onto Marc-145 cells. As shown in Figure 3D, the number of viral plaques in Xn + PRRSV cells was not obviously different from that in the DMSO + PRRSV control cells, indicating that Xn does not have a direct virucidal effect on PRRSV. Together, these data indicated that Xn inhibits PRRSV early in the infection process.

**Figure 4:**
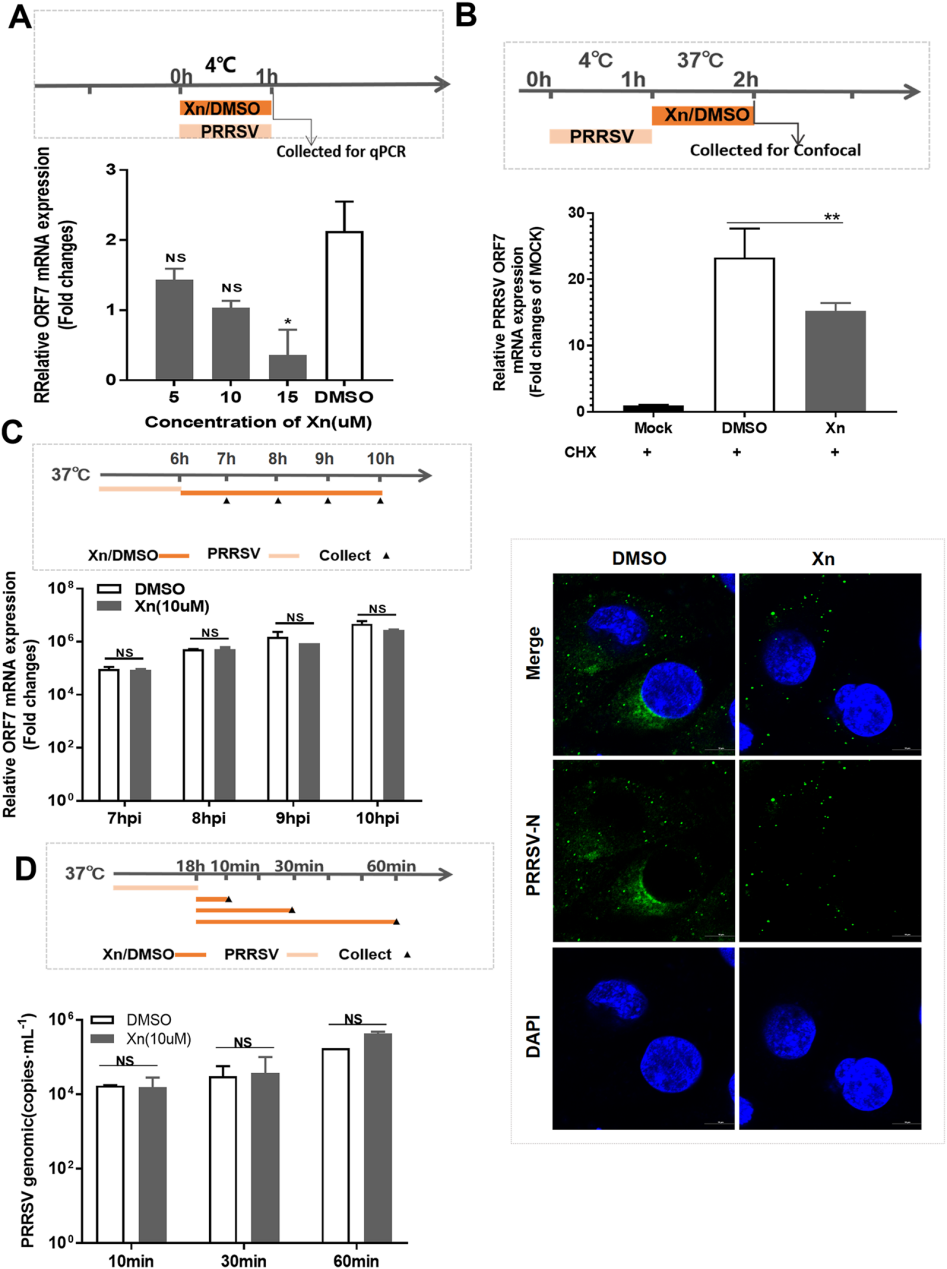
**Xn inhibits PRRSV adsorption and internalization in Marc-145 cells. A** Adsorption assay. Cells were incubated with a mixture of Xn or DMSO and PRRSV for 1 h at 4 °C and then harvested for qRT-PCR. **B** Internalization assay. Cells were treated with CHX (10 μg/mL) for 12 h first, then incubated with PRRSV (1 MOI) for 1 h at 4 °C, washed, and finally incubated with 10 µM Xn or DMSO for another 1 h at 37 °C. The levels of PRRSV ORF7 mRNA were detected by qRT-PCR, and the visible virions in cells were detected using IFA confocal laser-scanning microscopy. Scale bar 10 µm. **C** Cells were infected with PRRSV (1 MOI) for 6 h, and culture medium was replaced with 2% DMEM containing Xn (10 µM) or DMSO. At 7, 8, 9, and 10 hpi, the PRRSV ORF7 levels were measured using qRT-PCR. **D** Cells were infected with PRRSV (0.1 MOI) for 18 h, then washed and incubated with fresh DMEM containing 10 µM Xn or DMSO. Supernatants were harvested, and released virus was titred by plaque assay. qRT-PCR results are presented relative to the reference control (mock infected cells). GAPDH was used as the internal loading control. All assays were repeated at least three times, with each experiment performed in triplicate. Bars represent the means ± SEMs from three independent experiments. **P < 0.01; *P < 0.05 compared with the DMSO-treated group

To determine what stage of the PRRSV life cycle is specifically affected by Xn, Marc-145 cells were chilled at 4 °C for 1 h, and the culture supernatants were then replaced with a 4 °C mixture of Xn or DMSO and PRRSV (1 MOI). After incubation at 4 °C for another 1 h, the cells were washed and harvested for qRT-PCR of PRRSV ORF7 mRNA. The results showed that treatment with Xn resulted in reduced viral RNA detection in a dose-dependent manner, indicating that Xn affects PRRSV at the adsorption stage (Figure 4A). In a companion experiment, cells (pre-treated with 10 μg/mL CHX for 12 h) were incubated with PRRSV for 1 h at 4 °C (to allow virus to attach), washed, and then incubated in fresh DMEM-2% FBS containing 10 µM Xn for 2 h at 37 °C. The cells were probed for PRRSV N-protein and observed by confocal laser-scanning microscopy. The results showed that Xn inhibits PRRSV entry into cells even after the virus has attached to the cells (Figure 4B). In cells incubated with PRRSV for 6 h at 37 °C and then treated with Xn in fresh DMEM-2% FBS at 37 °C for an additional 1, 2, 3, and 4 h, qRT-PCR results showed that viral mRNA levels in the Xn-treated cells were not significantly different from those in the DMSO-treated control cells (Figure 4C). To explore the role of Xn in PRRSV release, Marc-145 cells were incubated with PRRSV for 24 h. The cells were washed and then incubated with fresh DMEM containing Xn for 10, 30, and 60 min. The virus titres in the supernatants from the Xn-treated cells were not significantly different than those in the supernatants from the DMSO-treated control cells (Figure 4D). Taken together, these results indicate that Xn inhibits PRRSV at the stages of adsorption and internalization.

### Transcriptional response of Marc-145 cells to xanthohumol treatment

**Figure 5:**
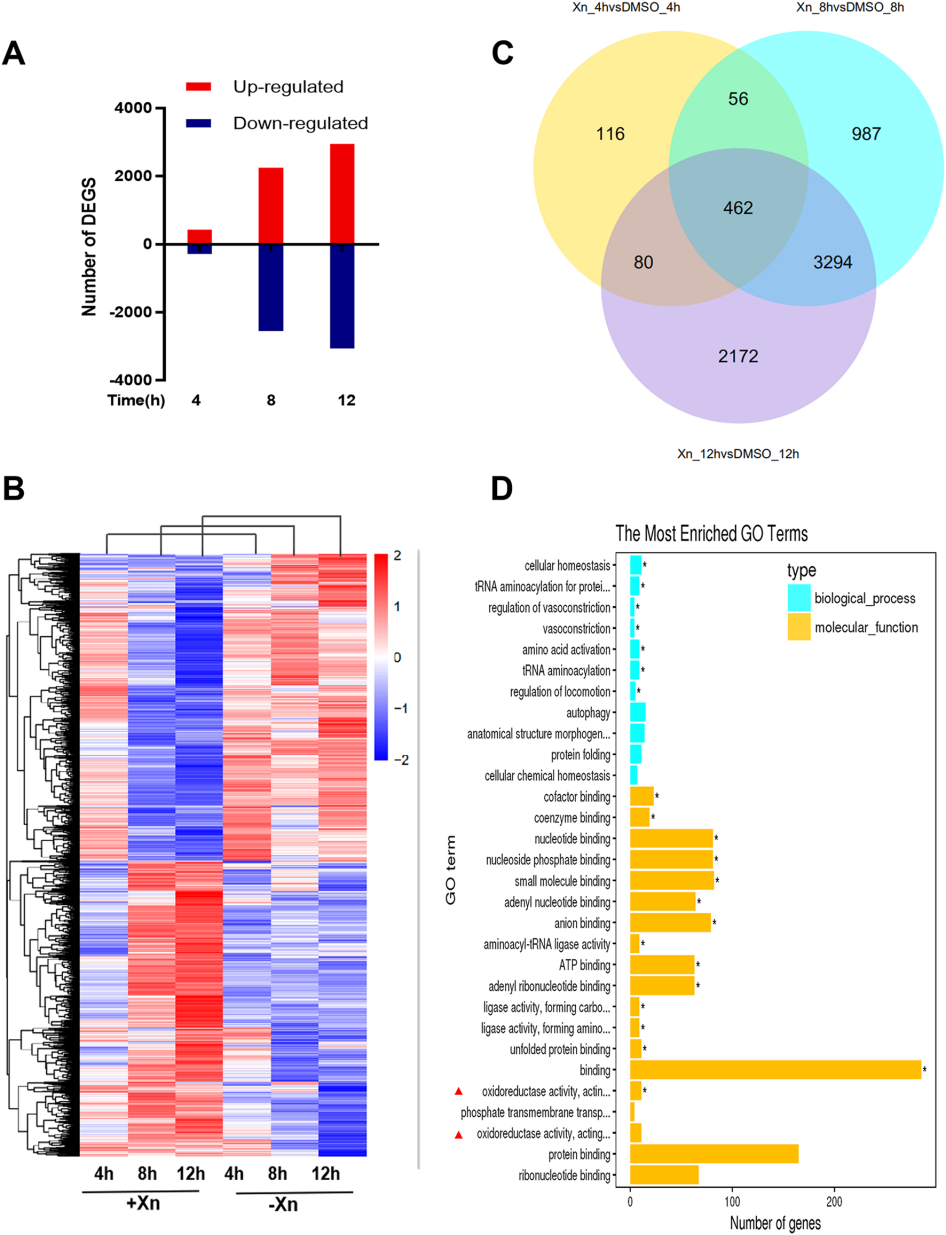
**Transcriptional response of Marc-145 cells to xanthohumol treatment.** RNA profiling by RNA sequencing of Marc-145 cells treated with 10 µM Xn or DMSO for 4, 8, and 12 h. **A** Waterfall plot of the up- and downregulated genes at each time point (DEGs selected based on padj < 0.05). **B** Unsupervised hierarchical cluster analysis of DEGs. DEG expression levels are represented as FPKM-normalized log_2_-transformed counts. Blue indicates low relative expression, and red indicates high relative expression. **C** Venn diagram of the unique and shared genes of each group. The cluster number for each component is listed. **D** Significantly enriched KEGG terms of the 462 shared genes

**Figure 6:**
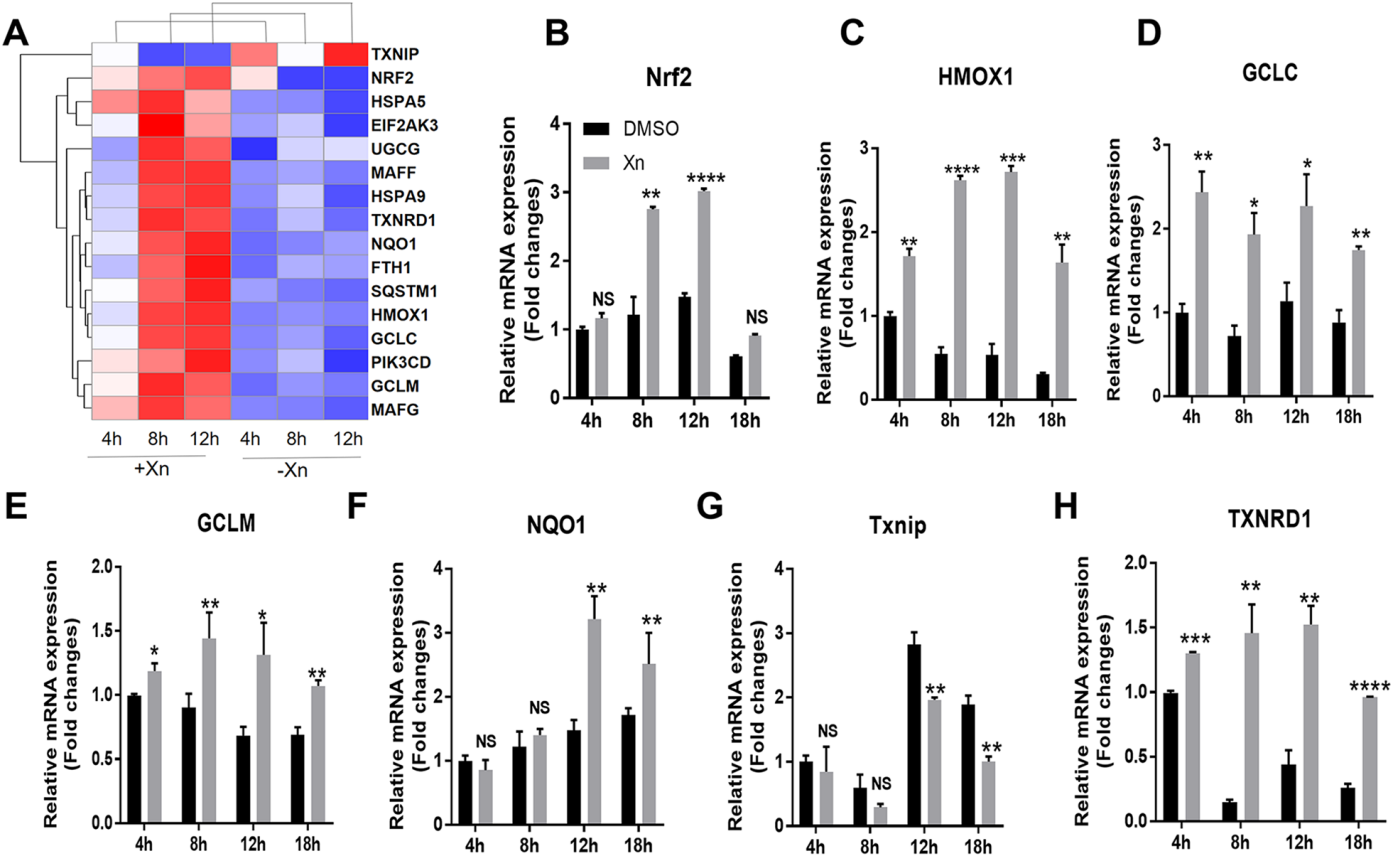
**Xanthohumol treatment upregulates the Nrf2 antioxidative pathway. A** Heat map of expressed Nrf2 antioxidant genes modulated by 10 µM Xn or DMSO treatment in Marc-145 cells. Red and blue correspond to relative up- and downregulation, respectively. **B**–**H** qRT-PCR quantification of the expression levels of Nrf2-regulated genes at four time points after 10 µM Xn treatment. The qRT-PCR was repeated at least three times, with each experiment performed in triplicate. ****P < 0.0001; ***P < 0.001; **P < 0.01; *P < 0.05 vs DMSO-treated cells for each time point

The effect of xanthohumol on the activation of the Nrf2 pathway in human hepatocellular carcinoma (HCC) HepG2 cells, immortalized human liver THLE-2 cells and mouse lung tissues [21, 22] has been reported. To investigate the mechanism underlying the anti-PRRSV effect of Xn, we used high-throughput RNA sequencing to examine gene expression in Marc-145 cells that had been treated with 10 µM Xn or DMSO for 4, 8, and 12 h. Figure 6A shows the numbers of differentially expressed genes (DEGs) that were identified at each time point, using the criteria of *p* < 0.05. We classified a gene as upregulated if its transcript was more abundant in cells treated with Xn than in cells treated with DMSO. The number of DEGs increased with treatment time, and after 12 h, 2493 genes were upregulated, and 3065 downregulated. In total, 462 DEGs appeared at all three sampled times (Figures 5B and C). GO analysis of the DEGs showed that the most enriched molecular functions were oxidoreductase activity and oxidation–reduction processes, as marked by asterisks (Figure 5D). GO analysis of the top 100 DEGs according to log2 fold change at 8 h suggested that oxidoreductase activity and the oxidoreductase complex were activated at this time point (Additional file 1). We therefore investigated the networks associated with oxidoreductase and the antioxidant response. Further analysis identified a large set of genes associated with the antioxidant response, including Nrf2, HMOX1, GCLC, GCLM, and NQO1 (Figure 6A). These genes, which are all part of the Nrf2 signalling pathway, are transcriptionally activated through mainly binding of Nrf2 to antioxidant response elements (AREs) at their 5′ promoters [23].

To confirm the results of the RNA-seq analysis, a qRT-PCR assay was conducted to measure the expression of *Nrf2*, *HMOX1*, *GCLC*, *GCLM*, *NQO1*, *TXNIP*, and *TXNRD1* in Xn-treated cells. The results (Figures 6B–H) showed that Xn treatment increased the expression of all 7 genes, consistent with the RNA-seq data. Taken together, these data indicate that Xn may protect against PRRSV infection by activating the host antioxidant response via stimulation of the Nrf2 signalling pathway.

### Xanthohumol upregulates the Nrf2 antioxidative pathway, inhibiting PRRSV proliferation

**Figure 7:**
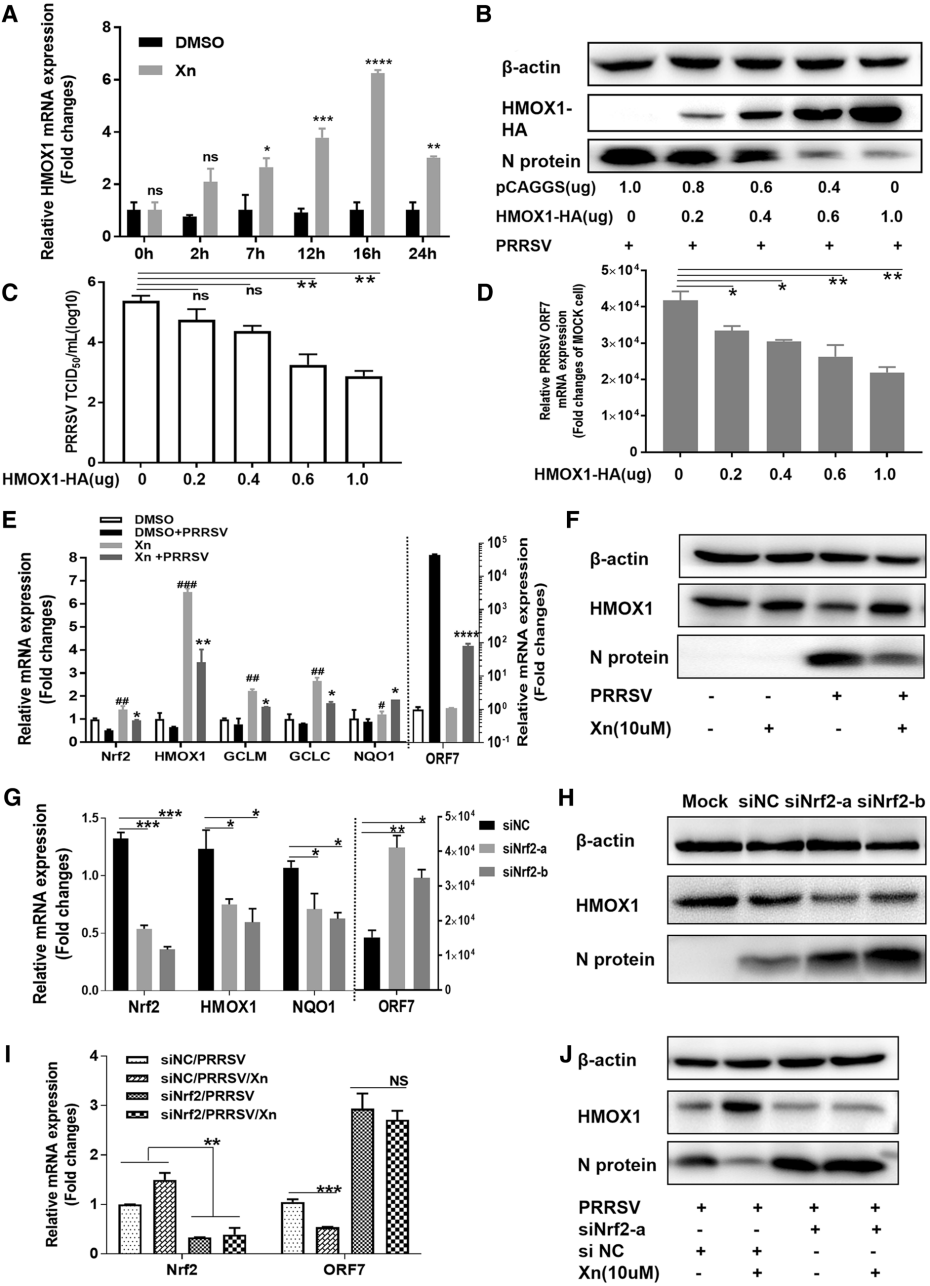
**Xanthohumol upregulates Nrf2–HMOX1 to inhibit PRRSV proliferation. A** Marc-145 cells were treated with 10 µM Xn for different times over 24 h, followed by qRT-PCR for HMOX1 mRNA. ***P < 0.001; **P < 0.01; *P < 0.05 vs DMSO-treated cells. **B**–**D** PRRSV replication in Marc-145 cells transfected with the indicated doses of pCAGGS-HMOX1 for 24 h and then infected with PRRSV (0.01 MOI) for 48 h was evaluated by Western blotting, TCID_50_ and qPCR. **E** Marc-145 cells were treated with Xn or DMSO for 1 h, then infected with PRRSV (0.1 MOI) for 1 h at 37 °C and finally cultured in medium containing 10 µM Xn or DMSO. Total RNA was extracted from lysates of cells at 24 hpi, and the mRNA levels of PRRSV ORF7 and a set of Nrf2-regulated antioxidant genes were detected by qRT-PCR. ^###^*P* < 0.001; ^##^*P* < 0.01; ^#^*P* < 0.05 vs DMSO-treated cells. ***P < 0.001; **P < 0.01; *P < 0.05 vs DMSO + PRRSV cells. **F** Cells were lysed and samples were prepared for Western blotting. **G** qRT-PCR and **H** Western blot analysis were performed on Marc-145 cells transfected with 50 pmol of siNC or Nrf2 siRNAs (siNrf2-a and siNrf2-b) using 5 µL of Lipofectamine RNAiMAX transfection reagent for 24 h. Cells were then infected with PRRSV (0.01 MOI), and after 36 h, cells were harvested for assays. **I** qRT-PCR and **J** Western blot analysis were performed on Marc-145 cells transfected with siNrf2-a and siNC. After 24 h, cells were treated with 10 µM Xn or DMSO for 1 h and then infected with PRRSV (0.01 MOI). After 36 h, cells were harvested for assays. All results are means ± standard deviations from three independent experiments performed in triplicate. ***P < 0.001; **P < 0.01; *P < 0.05 compared with the control group

HMOX1 (haem oxygenase 1) was highly upregulated at all three points in the RNA-seq analysis; it is a rate-limiting enzyme in haem degradation, has cytoprotective effects against oxidative stress, and plays an important role in the resolution of inflammation [24]. To examine the role of Xn-induced HMOX1 expression in PRRSV suppression, Marc-145 cells were treated with 10 µM Xn for different times over 24 h, followed by qRT-PCR for *HMOX1* mRNA. As shown in Figure 7A, *HMOX1* was induced by Xn in cells as a function of time until late in the experiment when induction levels fell sharply, indicating that Xn provides a continuous stimulus on *HMOX1.* We therefore investigated the role that HMOX1 plays in the protective effect of Xn against PRRSV in Marc-145 and PAM cells. Marc-145 cells, when 70% confluent, were transfected with different doses of pCAGGS-HMOX1 and pCAGGS and then infected with PRRSV (MOI 0.01). At 48 hpi, the levels of viral N-protein and HMOX1 in the cell lysates were examined by Western blotting. The levels of viral ORF7 mRNA and titres were detected by qRT-PCR and TCID_50_ assays. As expected, overexpression of HMOX1 in Marc-145 cells significantly inhibited PRRSV replication in a dose-dependent manner (Figures 7B and D). These results indicated that Xn inhibition of PRRSV is related to HMOX1.

*HMOX1* transcription is modulated by an intertwined circuit in which nuclear factor E2-related factor 2 (Nrf2) plays an essential role [25]. To examine the role of Nrf2 in the action of Xn against PRRSV infection, Marc-145 cells were infected with PRRSV (0.1 MOI) for 1 h at 37 °C and then cultured in medium containing 10 µM Xn or DMSO for 24 h. Total RNA was extracted from virus-infected cell lysates, and PRRSV ORF7 mRNA levels and the levels of a set of Nrf2-regulated antioxidant genes were quantified by qRT-PCR. As shown in Figure 7E, the expression of these Nrf2-regulated genes was downregulated slightly by PRRSV infection but significantly induced by Xn treatment. Infected cells treated with Xn had elevated mRNA expression of Nrf2 and its target genes, as well as decreased virus replication. Because we did not have a suitable Nrf2 antibody for detecting endogenous Nrf2 protein in Marc-145 cells, we used HMOX1 as an indicator of Nrf2 levels. As shown in Figure 7F, along with the decrease in N-protein, in PRRSV-infected cells treated with Xn, there was an accompanying increase in HMOX1. Xn treatment significantly inhibited PRRSV replication. In addition, Marc-145 cells were transfected with siRNAs against Nrf2 or with a nonspecific siRNA control (NC). After incubation for 24 h, cells were infected with PRRSV (0.01 MOI). At 36 hpi, cells were harvested for qRT-PCR and Western blotting. The results showed that the siRNAs siNrf2-a and siNrf2-b efficiently knocked down Nrf2 mRNA and protein levels (Figures 7G and H) and resulted in a significant increase in PRRSV replication. These results indicate that Nrf2 is a crucial protein involved in the host defence mechanism against PRRSV.

Finally, to identify the role of Nrf2 in the anti-PRRSV effect of Xn, Marc-145 cells were transfected with siNrf2-a or siNC. After 24 h, the cells were treated with 10 µM Xn for 1 h and then infected with PRRSV (0.01 MOI). At 36 hpi, cells were harvested for qRT-PCR and Western blotting. As expected, Xn significantly decreased PRRSV ORF7 mRNA and N-protein levels in cells transfected with siNC. However, in cells transfected with siNrf2, Xn had no inhibitory effect on PRRSV infection (Figures 7I and J). These results suggest that Nrf2 could be an effective target against PRRSV and that the anti-PRRSV action of Xn depends on its activation of Nrf2 and the Nrf2-regulated antioxidant pathway.

### Xn alleviates PRRSV-stimulated oxidative stress

**Figure 8:**
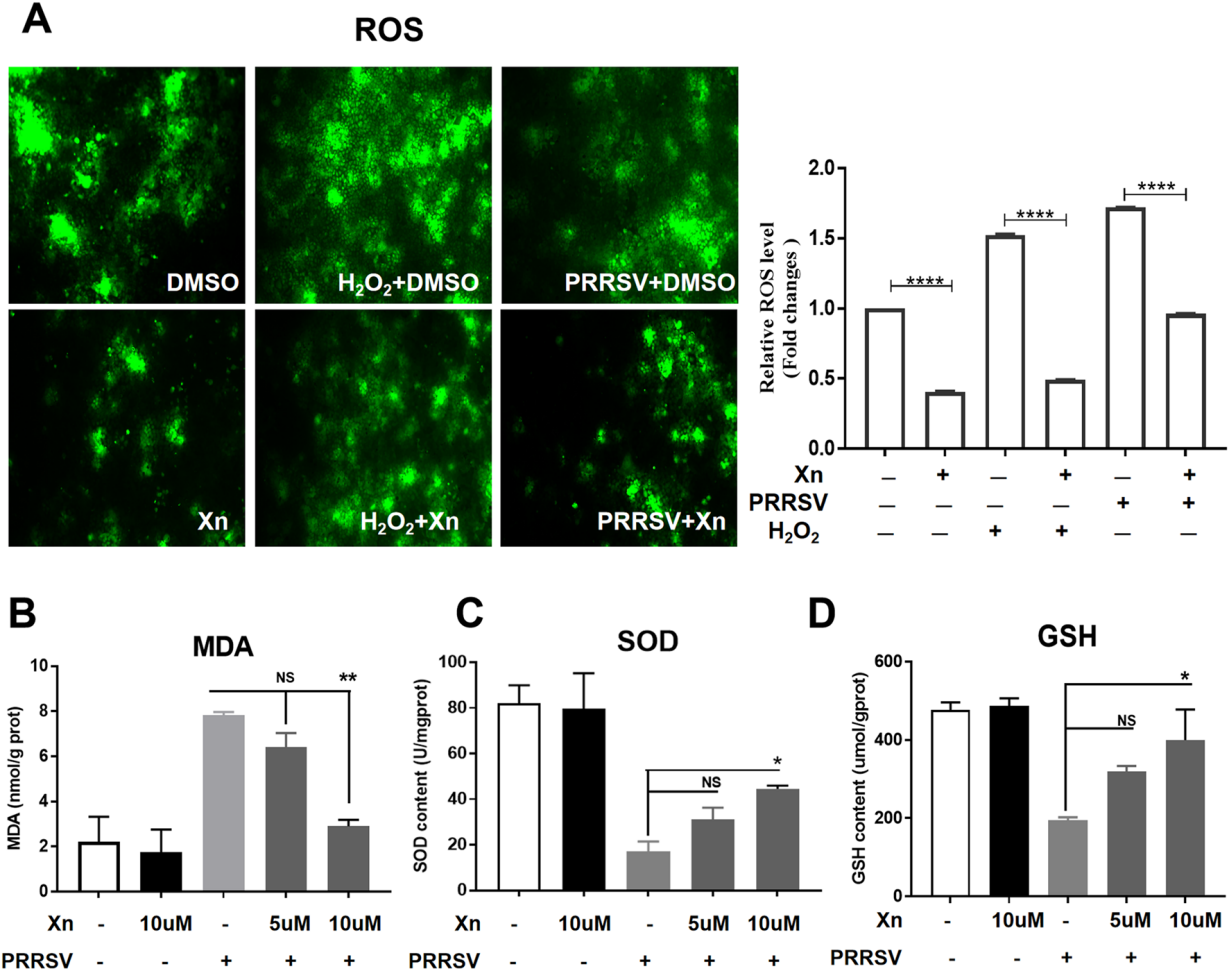
**Xanthohumol suppresses oxidative stress induced by PRRSV in Marc-145 cells. A** ROS generation in Marc-145 cells was determined using dichlorofluorescein (DCF), and images were acquired using an inverted fluorescence microscope (Nikon). ImageJ was used to quantify the intensity of the green fluorescence in the pictures. **B**–**D** Effects of Xn on MDA, SOD and GSH levels in PRRSV-infected Marc-145 cells. All results are means ± standard deviations from three independent experiments performed in triplicate. ***P < 0.001; **P < 0.01; *P < 0.05 compared with the PRRSV-infected cells

In Marc-145 cells, PRRSV infection induces oxidative stress in cells by generating reactive oxygen species (ROS), which play a key role in PRRSV pathogenesis [26]. Because of our transcriptome data regarding Xn-induced oxidoreductase activity and the Nrf2-regulated antioxidant genes and because Nrf2 plays a crucial role in oxidative stress [27], we explored further whether the inhibitory activity of Xn against PRRSV infection is related to its antioxidant activity. Marc-145 cells pre-treated with 12 µM H_2_O_2_ for 3 h and then incubated with Xn produced less ROS than untreated cells. IFA results showed that PRRSV infection resulted in increased ROS, and Xn treatment reduced ROS in infected and uninfected cells (Figure 8A). MDA, a product of lipid peroxidation and a biomarker for estimating oxidative stress, was also dramatically reduced by Xn treatment (Figure 8B). Furthermore, the reduction in SOD and glutathione peroxidase (GSH) as a result of PRRSV infection was alleviated by Xn (Figures 8C and D). These data indicate that Xn restores the oxidation balance in Marc-145 cells.

### Xanthohumol upregulates the Nrf2–HMOX1 antioxidative axis to inhibit PRRSV proliferation in PAMs

**Figure 9:**
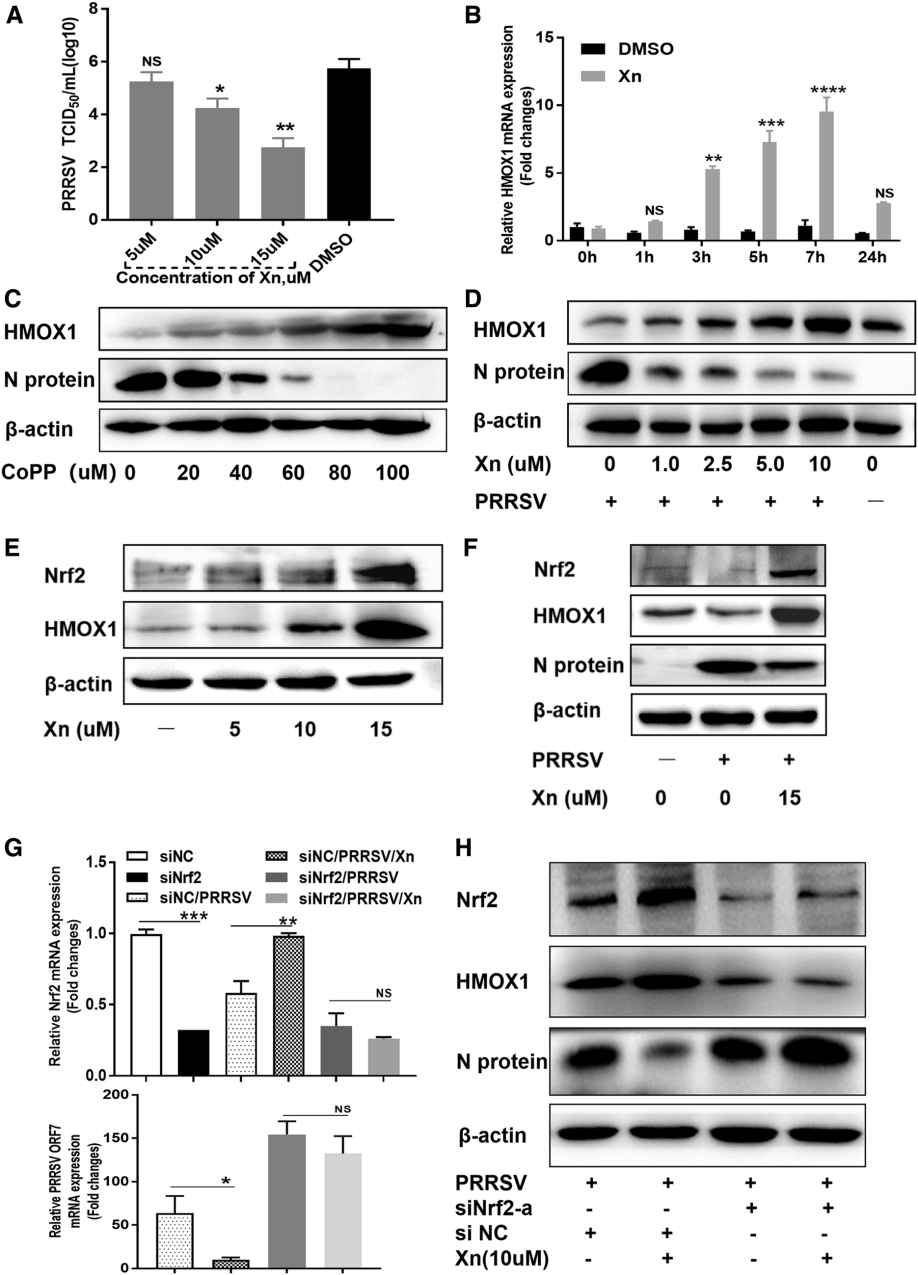
**Xanthohumol upregulates the Nrf2–HMOX1 antioxidative axis to inhibit PRRSV proliferation in PAMs. A** PAMs were pre-treated with DMSO or concentrations of Xn for 1 h and then infected with PRRSV (0.10 MOI) for 1 h at 37 °C. Cells were washed and incubated in fresh medium containing DMSO or Xn. At 24 hpi, cells were harvested for the viral titre. **B** PAMs were treated with 10 µM Xn for different times over 24 h, followed by qRT-PCR for HMOX1 mRNA. ***P < 0.001; **P < 0.01; *P < 0.05 vs DMSO-treated cells. **C** PAMs treated with CoPP (an inducer of HMOX1) for 6 h before PRRSV infection were examined for N-protein and endogenous HMOX1 by Western blotting. **D** PAMs were treated with an increasing multiplicity of Xn (0–10 µM) or DMSO for 1 h and then infected with PRRSV (MOI 0.01) for 1 h. After washing three times with PBS, culture medium containing Xn (0–10 µM) or DMSO was added back to the wells. At 24 hpi, the levels of HMOX1 and PRRSV N-protein were detected by Western blotting. **E** PAMs were treated with different doses of Xn for 24 h, and Nrf2 and HMOX1 were detected by Western blotting. **F** PAMs were treated with 15 µM Xn or DMSO for 1 h and then infected with PRRSV (MOI 0.01) for 1 h. After washing three times with PBS, culture medium containing 15 µM Xn or DMSO was added back to wells. At 24 hpi, the levels of Nrf2, HMOX1 and PRRSV N-protein were detected by Western blotting. **G**, **H** PAMs were transfected with 50 pmol of siRNA targeting Nrf2. After 48 h, they were treated with 10 µM Xn or DMSO for 1 h and then infected with PRRSV (0.01 MOI). After 36 hpi, the mRNA levels of Nrf2 and PRRSV ORF7 were detected by qRT-PCR (**G**). Meanwhile, the levels of Nrf2, HMOX1 and PRRSV N-protein were detected by Western blotting (**H**). ***P < 0.001; **P < 0.01; *P < 0.05 compared with the control group

Given that PAMs are natural target cells in pigs, we identified the role of Xn in PRRSV-infected PAMs. First, PAMs were treated with DMSO or 5, 10, and 15 µM Xn for 1 h at 37 °C and then infected with PRRSV (MOI 0.1) for 1 h. Cells were washed and incubated in fresh culture medium containing 5, 10, and 15 µM Xn for 24 h. Virus was detected by virus titration. As shown in Figure 9A, Xn reduced the PRRSV titre in a dose-dependent manner in PAMs, which is consistent with the results in Marc-145 cells. Then, we sought to identify the correlation between Xn and the Nrf2–HMOX1 axis in PAMs. PAMs were treated with 10 µM Xn for different times over 24 h, followed by qRT-PCR for HMOX1 mRNA. Similar to in Marc-145 cells, HMOX1 mRNA was also induced by Xn in PAMs (Figure 9B). We also used different concentrations of CoPP to treat PAMs for 6 h, then washed them 3 times and infected with PRRSV (MOI 0.01). At 48 hpi, the levels of viral N-protein and HMOX1 in the cell lysates were examined by Western blotting. As expected, stimulating the expression of endogenous HMOX1 in PAMs significantly inhibited PRRSV replication in a dose-dependent manner (Figure 9C). Similarly, Xn induced HMOX1 and then inhibited PRRSV in a dose-manner in PAMs (Figure 9D).

To further explore the role of Nrf2 in this process, PAMs were treated with Xn (5, 10, and 15 µM) and showed a dose-dependent increase in Nrf2 and HMOX1 (Figure 9E). We next treated PAMs with 15 µM Xn for 1 h and then infected them with PRRSV (MOI 0.01) for 1 h. After washing the cells with PBS, culture medium containing Xn was added back. At 24 hpi, the cells were harvested for Western blotting. As shown in Figure 9F, PRRSV infection suppressed Nrf2 and HMOX1 expression, but Xn treatment resulted in increased Nrf2 and HMOX1 expression and inhibition of PRRSV proliferation. Furthermore, Nrf2 expression knock-down also increased PRRSV replication in PAMs, and Xn had no inhibitory effect on PRRSV infection in PAMs transfected with the siRNA targeting Nrf2 (Figures 9G and H). These data support that the restrictive features of Xn on PRRSV replication depend on the Nrf2–HMOX1 axis.

**Figure 10:**
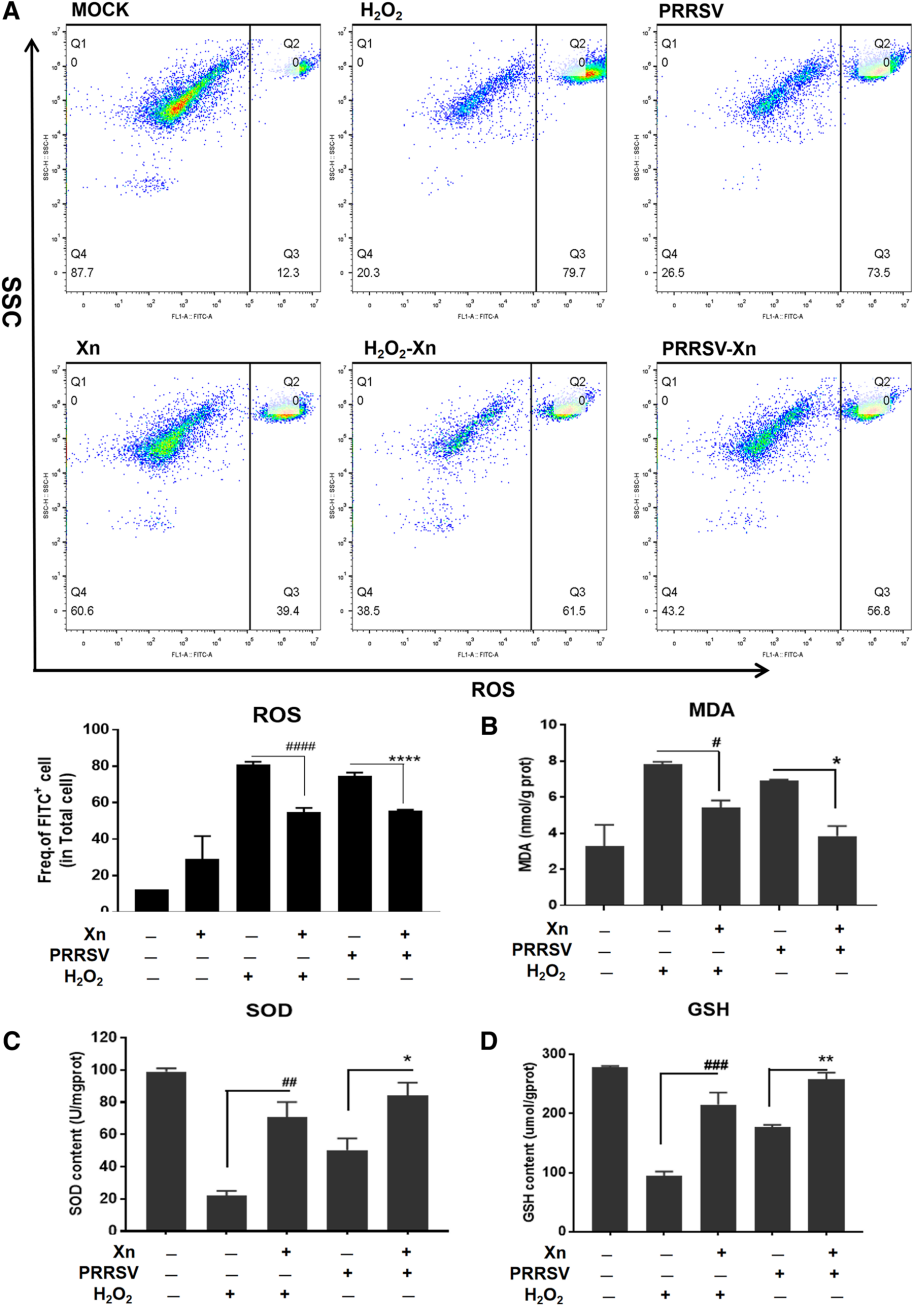
**Xanthohumol suppresses oxidative stress induced by PRRSV in PAMs. A** ROS generation in PAMs was determined using flow cytometry. The frequency of the cells that tested FITC positive among the total cells is shown in the lower right corner of the image. The histogram was used to quantify the number of FITC-positive cells in two independent trials. **B**–**D** Effects of 10 µM Xn on the levels of SOD, MDA, and GSH in PAMs infected with PRRSV or treated with H_2_O_2_. All results are means ± standard deviations from three independent experiments performed in triplicate. ^###^*P* < 0.001; ^##^*P* < 0.01; ^#^*P* < 0.05 vs H_2_O_2_-treated group. ***P < 0.001; **P < 0.01; *P < 0.05 vs PRRSV-infected cells

Given that Xn could alleviate PRRSV-stimulated oxidative stress in Marc-145 cells, we also tested whether this effect occurred in PAMs. PAMs pre-treated with 12 µM H_2_O_2_ for 3 h then incubated with 5 µM Xn produced less ROS than untreated cells. FACS analysis also showed that in PAMs, PRRSV infection resulted in increased ROS levels and that Xn treatment reduced ROS levels (Figure 10A); the raw flow cytometry data are shown in Additional file 2. The reduction in SOD and GSH levels as a result of PRRSV infection was alleviated by Xn (Figures 10B and C). The elevated MDA levels in infected cells were reduced by Xn (Figure 10D).

## Discussion

Natural products are a proven source of compounds for drug discovery, and the therapeutic use of plants has been known for millennia [28–30]. Natural compounds and compositions have also been a rich source for drugs against many viruses. Ouabain extracted from the seeds of *Strophanthus gratus*, bufalin isolated from the venom of the Bufo toad, and valinomycin extracted from *Streptomyces fulvissimus* can inhibit PRRSV replication at micromolar concentrations in Marc-145 cells [31]. Sodium tanshinone IIA sulfonate extracted from *Salvia miltiorrhiza* inactivates PRRSV directly, thereby inhibiting replication in vitro [16]. Glycyrrhizin extracted from liquorice root inhibits penetration of PRRSV but has little effect on viral adsorption or release in Marc-145 cells [32]. The mechanisms involved in these activities are not deeply understood. In this study, we screened a library of 386 natural products for anti-PRRSV activity. Xanthohumol significantly inhibited PRRSV proliferation in Marc-145 cells and PAMs and weakened PRRSV-induced oxidative stress by activating the Nrf2–HMOX1 pathway. It has promising therapeutic potential against PRRSV infection.

Xn is a prenylated flavonoid from the hop plant *Humulus lupulus* L [33]. Xn has anti-proliferative activity on breast, colon, and ovarian cancer cell lines [34]. Additionally, Xn has an anti-inflammatory effect on LPS-induced acute lung injury in mice [27], carbon tetrachloride-induced acute liver injury [35], and ischaemia reperfusion-induced liver injury [36]. Furthermore, Xn prevents cholesterol accumulation via *CETP* inhibition, leading to apoE upregulation in mice [37]. These research results demonstrate that Xn has a wide range of biological activities. We performed a transcriptome analysis of Xn-treated Marc-145 cells to initiate investigation into the mechanism of Xn inhibition of PRRSV. We found an enrichment of pathways associated with the Nrf2 antioxidant stress response; among them, *HMOX1* was the most highly stimulated by Xn treatment. Haem oxygenase catabolizes haem and has three isoforms; *HMOX1* is a rate-limiting enzyme in haem catabolism, leading to the generation of bilirubin, free iron, and carbon monoxide. *HMOX1* is a stress response protein that can be induced by various oxidative agents through *Nrf2*-mediated transcriptional activation of AREs [38]. It can maintain redox homeostasis, cooperating with other antioxidant enzymes. HMOX1 has also been shown to have significant antiviral effects [39–41]. We found that Xn could induce the expression of *HMOX1* in Marc-145 cells and PAMs in a dose-dependent manner. Overexpressed or induced *HMOX1* attenuated PRRSV replication in Marc-145 and PAM cells. Nuclear factor-erythroid 2-related factor 2 (*Nrf2*) is an essential factor in the cell’s defence system, primarily regulating the expression of many genes involved in the oxidant response, such as *HMOX1, NQO1, GCLC,* and *GCLM* [42]. Transcriptional activation of these enzymes is through mainly binding of Nrf2 to antioxidant response elements (AREs) on their 5′ promoter [43, 44]. The protective role of the Nrf2-ARE pathway has been examined in experimental models of various inflammatory and oxidative stress-induced respiratory diseases. For example, Nrf2 via the Nrf2-mediated signalling pathway limits oxidative stress and weakens the severity of allergic airway inflammation caused by asthma [45]. The importance of the Nrf2 pathway in viral pathogenesis was demonstrated in a study involving Nrf2 knockout mice. In that study, mice challenged with respiratory syncytial virus or influenza virus had elevated levels of viral replication and increased inflammatory responses and injury in their lungs [46]. Activation of the antioxidant pathways regulated by Nrf2 supports the regulation of antiviral and apoptotic responses by maintaining redox homeostasis in dengue virus-infected dendritic cells [47]. In this study, we found that Nrf2 had antiviral activity against PRRSV infection in Marc-145 cells and PAMs and that Xn directly targeted Nrf2 and stimulated the antioxidant pathways regulated by *Nrf2.* These results indicated that the Nrf2 antioxidant pathway plays a role in the anti-PRRSV activity of Xn. Nrf2 is a latent transcription factor present in an inactive form in the cytoplasm of cells as a complex with inhibitory proteins such as Keap1 (Kelch-like-associated protein1). Upon treatment with exogenous factors or induction of oxidative stress, Nrf2 is activated via the disruption of this complex, allowing the subsequent translocation of active Nrf2 to the nucleus, where it binds to ARE sequences to activate transcription of relevant inducible genes [48]. This mechanism is a very important aspect of Nrf2 biology and the cellular response to oxidative stress. It should be determined whether translocation of Nrf2 from the cytoplasm to the nucleus of cells occurs upon PRRSV infection, Xn treatment or both.

ROS generation is common in virus-infected cells. Cellular oxidative stress is significantly increased in dengue virus-infected dendritic cells [47]. Influenza virus infection increases reactive peroxynitrite formation and causes oxidative stress in lungs [49, 50], and PRRSV infection induces oxidative stress via ROS production in Marc-145 cells [51, 52]. The upregulation of ROS is related to mitochondrial dysfunction [52]. PRRSV downregulates the expression of superoxide dismutase, reduces that of glutathione, and increases the production of ROS and malondialdehyde in Marc-145 cells, PAMs, and the lung tissues of infected pigs. We found that Xn treatment effectively decreased PRRSV-induced oxidative stress. The Nrf2-mediated antioxidant response maintains an appropriate redox status and thus inhibits PRRSV-induced ROS damage.

The PRRSV life cycle can be divided into 4 basic stages: attachment, entry, replication and release [53, 54]. Our results showed that xanthohumol significantly inhibited PRRSV adsorption and entry in Marc-145 cells and PAMs. Numerous host cell-dependent factors can affect and control PRRSV attachment and uptake by (i) the level of a specific virus receptor, such as CD163; (ii) host-cell derived innate immune defence molecules aimed at binding and neutralizing the infectious virions; and (iii) antiviral mediators limiting viral replication [55]. Therefore, modification of any or several of these factors by Xn treatment could affect susceptibility to PRRSV infection. Here, our data demonstrated that siRNA-mediated knock-down of Nrf2 in MARC-145 cells increased PRRSV replication in these cells (Additional file 2). In addition, PRRSV infection induced oxidative stress. This result indicates that this virus-induced oxidative stress is beneficial for viral replication or that Nrf2 is involved in some way in the cellular innate antiviral response. In addition, we also noted that Xn downregulated the RNA expression of CD163, a key receptor of PRRSV [56] (Additional file 3). Nrf2 exists in most organs and affects the expression of nearly 500 proteins that act as redox balancing factors, detoxifying enzymes, stress response proteins and metabolic enzymes [57]. The changes in the cellular innate antiviral response, such as expression of RIG-I, IFN-β, and ISGs, and the receptor of PRRSV after treatment with Xn should be explored in the future.

**Figure 11:**
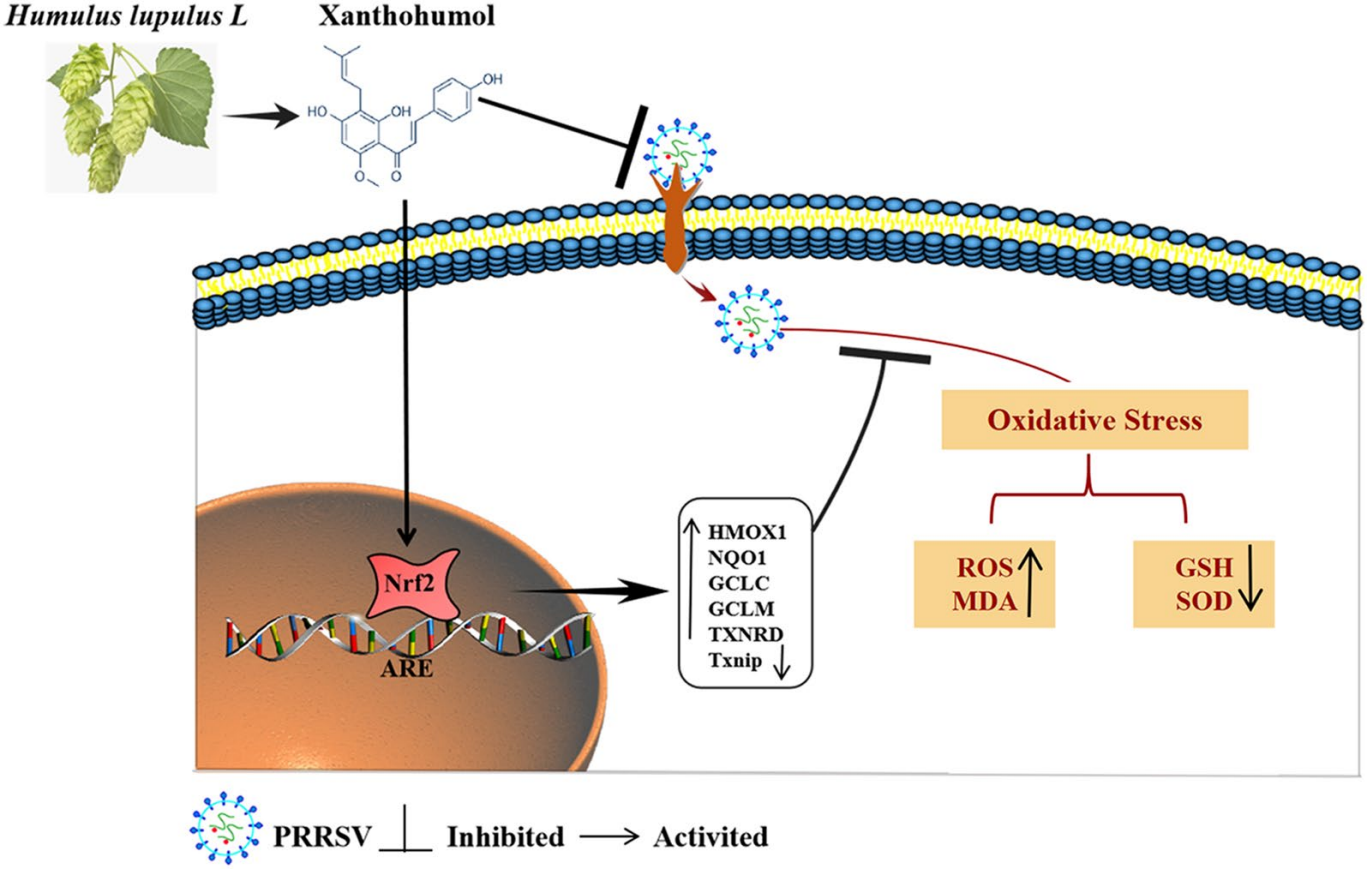
**Scheme summarizing the inhibitory effect of xanthohumol on PRRSV proliferation via activation of the Nrf2–HMOX1 axis.** Xanthohumol, a prenylated flavonoid found in hops, inhibits PRRSV adsorption onto and internalization into cells. Xn treatment also stimulates Nrf2, which causes an increase in the levels of the antioxidant enzymes HMOX1, GCLC, GCLM, and NQO1 and a suppression of oxidative stress, resulting in a decrease in virus proliferation

In conclusion, (Figure 11), xanthohumol, a natural product derived from hops, effectively attenuated PRRSV proliferation and suppressed oxidative stress induced by PRRSV. These activities of Xn occurred largely via the activation of the Nrf2–HMOX1 pathway. This finding offers new and promising therapeutic possibilities for combating infections caused by PRRSV.

## Supplementary information

**Additional file 1.** **GO analysis of the top 100 DEGs according to log2 fold change at three time points**. (A) GO analysis of the top 100 DEGs according to log2 fold change at 4 h. (B) GO analysis of the top 100 DEGs according to log2 fold change at 8 h. (C) GO analysis of the top 100 DEGs according to log2 fold change at 12 h.

**Additional file 2.** **Nrf2 knock-down blocked PRRSV entry**. Marc-145 cells were transfected with 50 pmol of siNrf2 or siNC. After 24 h, CHX (10 μg/mL) was added and incubated for 12 h, and then cells were incubated with PRRSV(1 MOI) for 1 h at 4 °C, washed, and incubated for another 1 h at 37 °C. The mRNA levels of PRRSV ORF7 and Nrf2 were detected by qRT-PCR. **P < 0.01; *P < 0.05 vs siNC group.

**Additional file 3.** **Xn treatment downregulated the CD163 mRNA levels in Marc-145 cells**. (A) Heat map of expressed genes modulated by 10 µM Xn or DMSO treatment in Marc-145 cells. Red and blue correspond to relative up- and downregulation, respectively. (B) Marc-145 cells were treated with 10 µM Xn for 4, 8, 12 and 18 h, followed by qRT-PCR for CD163 mRNA levels. **P < 0.01; *P < 0.05 vs DMSO-treated cells.

## References

[1] Lunney JK, Benfield DA, Rowland RR (2010). Porcine reproductive and respiratory syndrome virus: an update on an emerging and re-emerging viral disease of swine. Virus Res.

[2] Neumann EJ, Kliebenstein JB, Johnson CD, Mabry JW, Bush EJ, Seitzinger AH, Green AL, Zimmerman JJ (2005). Assessment of the economic impact of porcine reproductive and respiratory syndrome on swine production in the United States. J Am Vet Med Assoc.

[3] Nelsen CJ, Murtaugh MP, Faaberg KS (1999). Porcine reproductive and respiratory syndrome virus comparison: divergent evolution on two continents. J Virol.

[4] Tian K, Yu X, Zhao T, Feng Y, Cao Z, Wang C, Hu Y, Chen X, Hu D, Tian X, Liu D, Zhang S, Deng X, Ding Y, Yang L, Zhang Y, Xiao H, Qiao M, Wang B, Hou L, Wang X, Yang X, Kang L, Sun M, Jin P, Wang S, Kitamura Y, Yan J, Gao GF (2007). Emergence of fatal PRRSV variants: unparalleled outbreaks of atypical PRRS in China and molecular dissection of the unique hallmark. PLoS One.

[5] Zhang Q, Bai J, Hou H, Song Z, Zhao Y, Jiang P (2017). A novel recombinant porcine reproductive and respiratory syndrome virus with significant variation in cell adaption and pathogenicity. Vet Microbiol.

[6] Murtaugh MP, Genzow M (2011). Immunological solutions for treatment and prevention of porcine reproductive and respiratory syndrome (PRRS). Vaccine.

[7] Renukaradhya GJ, Xiang-Jin M, Calvert JG, Michael R, Lager KM (2015). Inactivated and subunit vaccines against porcine reproductive and respiratory syndrome: current status and future direction. Vaccine.

[8] Fang J, Wang H, Bai J, Zhang Q, Li Y, Liu F, Jiang P (2016). Monkey viperin restricts porcine reproductive and respiratory syndrome virus replication. PLoS One.

[9] Wang H, Bai J, Fan B, Li Y, Zhang Q, Jiang P (2016). The interferon-induced Mx2 inhibits porcine reproductive and respiratory syndrome virus replication. J Interferon Cytokine Res.

[10] Zhao J, Feng N, Li Z, Wang P, Qi Z, Liang W, Zhou X, Xu X, Liu B (2016). 2′,5′-Oligoadenylate synthetase 1(OAS1) inhibits PRRSV replication in Marc-145 cells. Antivir Res.

[11] Zhang L, Liu J, Bai J, Du Y, Wang X, Liu X, Jiang P (2013). Poly(I:C) inhibits porcine reproductive and respiratory syndrome virus replication in MARC-145 cells via activation of IFIT3. Antivir Res.

[12] Song Z, Zhang Q, Liu X, Bai J, Zhao Y, Wang X, Jiang P (2017). Cholesterol 25-hydroxylase is an interferon-inducible factor that protects against porcine reproductive and respiratory syndrome virus infection. Vet Microbiol.

[13] Ke W, Fang L, Jing H, Tao R, Wang T, Li Y, Long S, Wang D, Xiao S (2017). Cholesterol 25-hydroxylase inhibits porcine reproductive and respiratory syndrome virus replication through enzyme activity dependent and independent mechanisms. J Virol.

[14] Xie J, Zhou H, Cui J, Chen Y, Zhang M, Deng S, Zhou P, Su S, Zhang G (2014). Inhibition of porcine reproductive and respiratory syndrome virus by specific siRNA targeting Nsp9 gene. Infect Genet Evol.

[15] Li L, Li Q, Bao Y, Li J, Chen Z, Yu X, Zhao Y, Tian K, Li N (2014). RNAi-based inhibition of porcine reproductive and respiratory syndrome virus replication in transgenic pigs. J Biotechnol.

[16] Sun N, Zhao X, Bai XY, Niu L, Song MQ, Sun YG, Jiang JB, Li HQ (2012). Anti-PRRSV effect and mechanism of sodium tanshinone IIA sulfonate in vitro. J Asian Nat Prod Res.

[17] Zhang M, Wu Q, Chen Y, Duan M, Tian G, Deng X, Sun Y, Zhou T, Zhang G, Chen W, Chen J (2018). Inhibition of proanthocyanidin A2 on porcine reproductive and respiratory syndrome virus replication in vitro. PLoS One.

[18] Li L, Tian X, Chen J, Li P, Zheng Q, Hou J (2018). Griffithsin inhibits porcine reproductive and respiratory syndrome virus infection in vitro. Arch Virol.

[19] Ge M, Xiao Y, Chen H, Luo F, Du G, Zeng F (2018). Multiple antiviral approaches of (-)-epigallocatechin-3-gallate (EGCG) against porcine reproductive and respiratory syndrome virus infection in vitro. Antivir Res.

[20] Chen X, Bai J, Liu X, Song Z, Zhang Q, Wang X, Jiang P (2018). Nsp1α of porcine reproductive and respiratory syndrome virus strain BB0907 impairs the function of monocyte-derived dendritic cells via the release of soluble CD83. J Virol.

[21] Krajka-Kuźniak V, Paluszczak J, Baer-Dubowska W (2013). Xanthohumol induces phase II enzymes via Nrf2 in human hepatocytes in vitro. Toxicol In Vitro.

[22] Cui B, Zhang S, Wang Y, Guo Y (2019). Farrerol attenuates β-amyloid-induced oxidative stress and inflammation through Nrf2/Keap1 pathway in a microglia cell line. Biomed Pharmacother.

[23] Li Y, Zhao Y, Cheng M, Qiao Y, Wang Y, Xiong W, Yue W (2018). Suppression of microRNA-144-3p attenuates oxygen-glucose deprivation/reoxygenation-induced neuronal injury by promoting Brg1/Nrf2/ARE signaling. J Biochem Mol Toxicol.

[24] Pae HO, Lee YC, Chung HT (2008). Heme oxygenase-1 and carbon monoxide: emerging therapeutic targets in inflammation and allergy. Recent Pat Inflamm Allergy Drug Discov.

[25] Dennery PA (2014). Signaling function of heme oxygenase proteins. Antioxid Redox Signal.

[26] Guo R, Davis D, Fang Y (2018). Intercellular transfer of mitochondria rescues virus-induced cell death but facilitates cell-to-cell spreading of porcine reproductive and respiratory syndrome virus. Virology.

[27] Lv H, Liu Q, Wen Z, Feng H, Deng X, Ci X (2017). Xanthohumol ameliorates lipopolysaccharide (LPS)-induced acute lung injury via induction of AMPK/GSK3β-Nrf2 signal axis. Redox Biol.

[28] Hossain MK, Choi HY, Hwang JS, Dayem AA, Kim JH, Kim YB, Poo H, Cho SG (2014). Antiviral activity of 3,4, dihydroxyflavone on influenza a virus. J Microbiol.

[29] Efferth T (2018). Beyond malaria: the inhibition of viruses by artemisinin-type compounds. Biotechnol Adv.

[30] Dias DA, Urban S, Roessner U (2012). A historical overview of natural products in drug discovery. Metabolites.

[31] Karuppannan AK, Wu KX, Qiang J, Chu JJ, Kwang J (2012). Natural compounds inhibiting the replication of Porcine reproductive and respiratory syndrome virus. Antivir Res.

[32] Duan E, Wang D, Fang L, Ma J, Luo J, Chen H, Li K, Xiao S (2015). Suppression of porcine reproductive and respiratory syndrome virus proliferation by glycyrrhizin. Antiviral Res.

[33] Sousa LRFD, Wu H, Nebo L, Fernandes JB, Kiefer W, Kanitz M, Bodem J, Diederich WE, Schirmeister T (2015). Flavonoids as noncompetitive inhibitors of Dengue virus NS2B-NS3 protease: inhibition kinetics and docking studies. Bioorgan Med Chem.

[34] Miranda CL, Stevens JF, Helmrich A, Henderson MC, Rodriguez RJ, Yang YH, Deinzer ML, Barnes DW, Buhler DR (1999). Antiproliferative and cytotoxic effects of prenylated flavonoids from hops (*Humulus lupulus*) in human cancer cell lines. Food Chem Toxicol.

[35] Pinto C, Duque AL, Rodrguez-Galden B, Cestero JJ, Macas P (2012). Xanthohumol prevents carbon tetrachloride-induced acute liver injury in rats. Food Chem Toxicol.

[36] Dorn C, Massinger S, Wuzik A, Heilmann J, Hellerbrand C (2013). Xanthohumol suppresses inflammatory response to warm ischemia-reperfusion induced liver injury. Exp Mol Pathol.

[37] Hirata H, Yimin Segawa S, Ozaki M, Kobayashi N, Shigyo T, Chiba H (2012). Xanthohumol prevents atherosclerosis by reducing arterial cholesterol content via CETP and apolipoprotein E in CETP-transgenic mice. PLoS One.

[38] Ryter SW, Choi AM (2002). Heme oxygenase-1: molecular mechanisms of gene expression in oxygen-related stress. Antioxid Redox Signal.

[39] Protzer U, Seyfried S, Quasdorff M, Sass G, Svorcova M, Webb D, Bohne F, HöSel M, Schirmacher P, Tiegs G (2007). Antiviral activity and hepatoprotection by heme oxygenase-1 in hepatitis B virus infection. Gastroenterology.

[40] Lehmann E, El-Tantawy WH, Ocker M, Bartenschlager R, Lohmann V, Hashemolhosseini S, Tiegs G, Sass G (2010). The heme oxygenase 1 product biliverdin interferes with hepatitis C virus replication by increasing antiviral interferon response. Hepatology.

[41] Devadas K, Dhawan S (2006). Hemin activation ameliorates HIV-1 infection via heme oxygenase-1 induction. J Immunol.

[42] Suzuki T, Motohashi H, Yamamoto M (2013). Toward clinical application of the Keap1-Nrf2 pathway. Trends Pharmacol Sci.

[43] Mills EL, Ryan DG, Prag HA, Dikovskaya D, Menon D, Zaslona Z, Jedrychowski MP, Costa ASH, Higgins M, Hams E, Szpyt J, Runtsch MC, King MS, McGouran JF, Fischer R, Kessler BM, McGettrick AF, Hughes MM, Carroll RG, Booty LM, Knatko EV, Meakin PJ, Ashford MLJ, Modis LK, Brunori G, Sévin DC, Fallon PG, Caldwell ST, Kunji ERS, Chouchani ET (2018). Itaconate is an anti-inflammatory metabolite that activates Nrf2 via alkylation of KEAP1. Nature.

[44] Krajka-Kuźniak V, Paluszczak J, Baer-Dubowska W (2017). The Nrf2-ARE signaling pathway: an update on its regulation and possible role in cancer prevention and treatment. Pharmacol Rep.

[45] Rangasamy T, Guo J, Mitzner WA, Roman J, Singh A, Fryer AD, Yamamoto M, Kensler TW, Tuder RM, Georas SN, Biswal S (2005). Disruption of Nrf2 enhances susceptibility to severe airway inflammation and asthma in mice. J Exp Med.

[46] Cho H, Imani F, Miller-DeGraff L, Walters D, Melendi GA, Yamamoto M, Polack FP, Kleeberger SR (2009). Antiviral activity of Nrf2 in a murine model of respiratory syncytial virus disease. Am J Resp Crit Care.

[47] Olagnier D, Peri S, Steel C, van Montfoort N, Chiang C, Beljanski V, Slifker M, He Z, Nichols CN, Lin R, Balachandran S, Hiscott J (2014). Cellular oxidative stress response controls the antiviral and apoptotic programs in dengue virus-infected dendritic cells. PLoS Pathog.

[48] Ge M, Yao W, Yuan D, Zhou S, Chen X, Zhang Y, Li H, Xia Z, Hei Z (2017). Brg1-mediated Nrf2/HO-1 pathway activation alleviates hepatic ischemia-reperfusion injury. Cell Death Dis.

[49] Vlahos R, Stambas J, Selemidis S (2012). Suppressing production of reactive oxygen species (ROS) for influenza A virus therapy. Trends Pharmacol Sci.

[50] Choi AM, Knobil K, Otterbein SL, Eastman DA, Jacoby DB (1996). Oxidant stress responses in influenza virus pneumonia: gene expression and transcription factor activation. Am J Physiol.

[51] Lee S, Kleiboeker SB (2005). Porcine arterivirus activates the NF-κB pathway through IκB degradation. Virology.

[52] Sang-Myeong L, Kleiboeker SB (2007). Porcine reproductive and respiratory syndrome virus induces apoptosis through a mitochondria-mediated pathway. Virology.

[53] Kappes MA, Faaberg KS (2015). PRRSV structure, replication and recombination: origin of phenotype and genotype diversity. Virology.

[54] Wang HM, Liu TX, Wang TY, Wang G, Liu YG, Liu SG, Tang YD, Cai XH (2018). Isobavachalcone inhibits post-entry stages of the porcine reproductive and respiratory syndrome virus life cycle. Arch Virol.

[55] Wang R, Wang X, Ni B, Huan CC, Wu JQ, Wen LB, Liao Y, Tong GZ, Ding C, Fan HJ, Mao X (2016). Syndecan-4, a PRRSV attachment factor, mediates PRRSV entry through its interaction with EGFR. Biochem Biophys Res Commun.

[56] Burkard C, Lillico SG, Reid E, Jackson B, Mileham AJ, Ait-Ali T, Whitelaw CBA, Archibald AL (2017). Precision engineering for PRRSV resistance in pigs: macrophages from genome edited pigs lacking CD163 SRCR56 domain are fully resistant to both PRRSV genotypes while maintaining biological function. PLoS Pathog.

[57] Liu T, Liu M, Liu S, Chen F, Chen F, Yang J (2017). The role of oxidative stress in influenza virus infection. Microbes Infect.

