# Supplementary figures and images for: Xanthohumol inhibits PRRSV proliferation and alleviates oxidative stress induced by PRRSV via the Nrf2–HMOX1 axis
Source: Vet Res. 2019 Sep 11;50:61. doi: 10.1186/s13567-019-0679-2 (PMC6737628; doi:10.1186/s13567-019-0679-2)

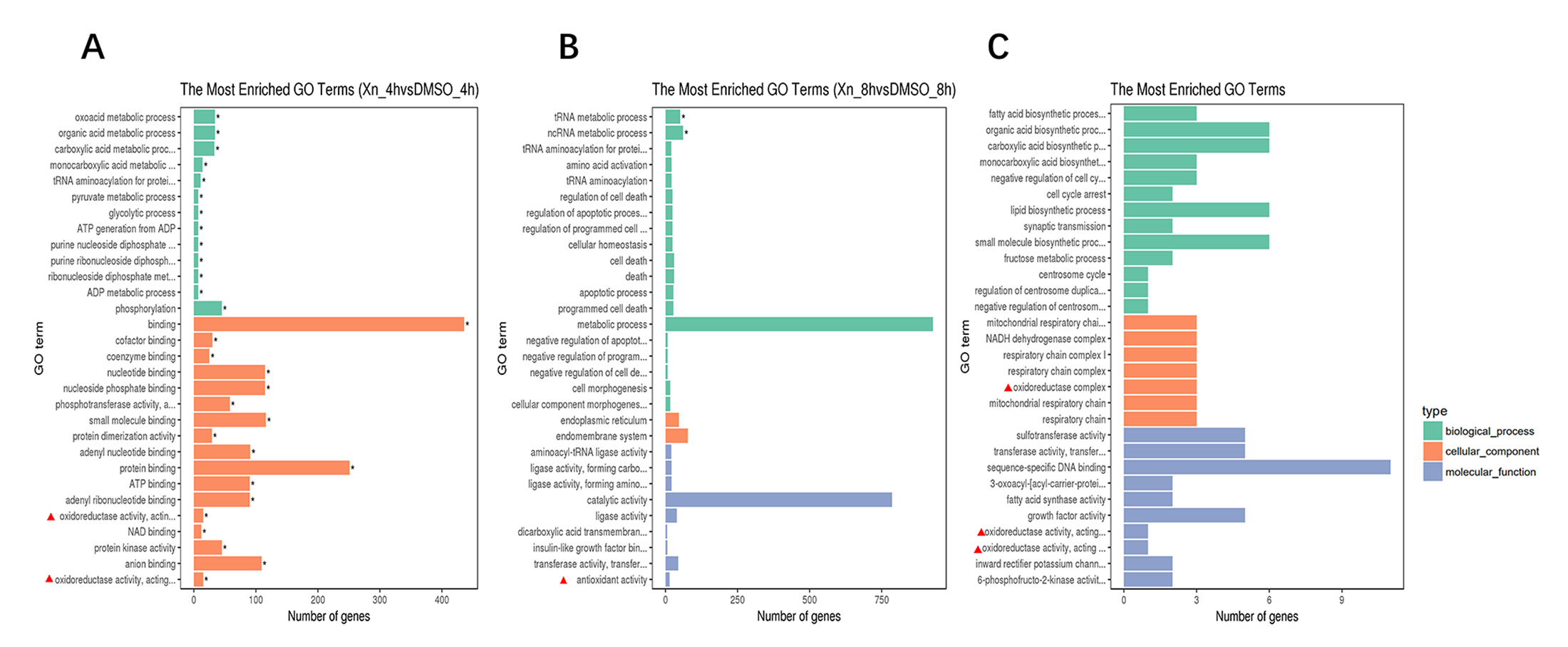

Supplement: Supplementary file 1 — Additional file 1. GO analysis of the top 100 DEGs according to log2 fold change at three time points. (A) GO analysis of the top 100 DEGs according to log2 fold change at 4 h. (B) GO analysis of the top 100 DEGs according to log2 fold change at 8 h. (C) GO analysis of the top 100 DEGs according to log2 fold change at 12 h. [file 13567_2019_679_MOESM1_ESM.tif]

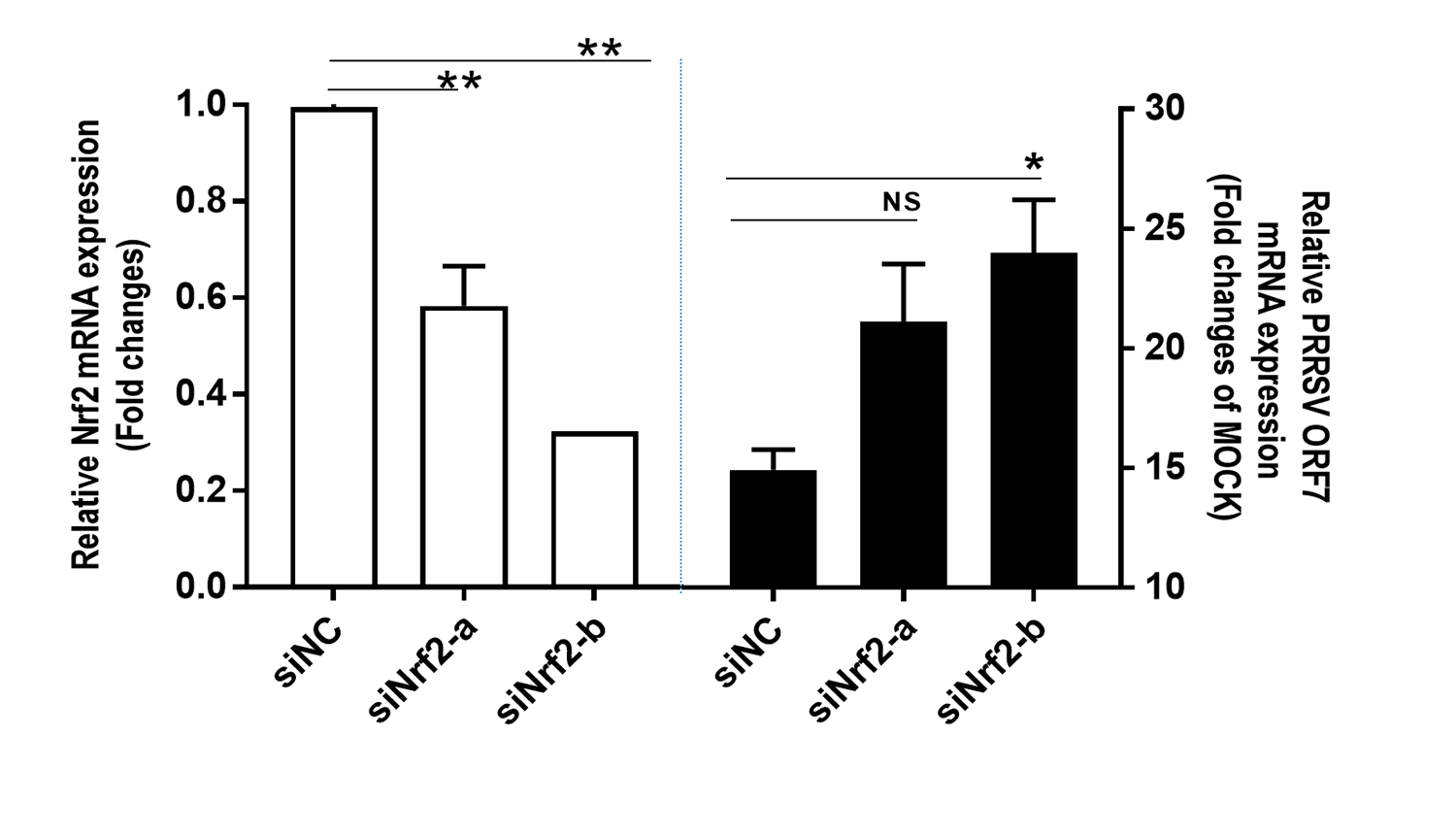

Supplement: Supplementary file 2 — Additional file 2. Nrf2 knock-down blocked PRRSV entry. Marc-145 cells were transfected with 50 pmol of siNrf2 or siNC. After 24 h, CHX (10 μg/mL) was added and incubated for 12 h, and then cells were incubated with PRRSV(1 MOI) for 1 h at 4 °C, washed, and incubated for another 1 h at 37 °C. The mRNA levels of PRRSV ORF7 and Nrf2 were detected by qRT-PCR. **P < 0.01; *P < 0.05 vs siNC group. [file 13567_2019_679_MOESM2_ESM.tif]

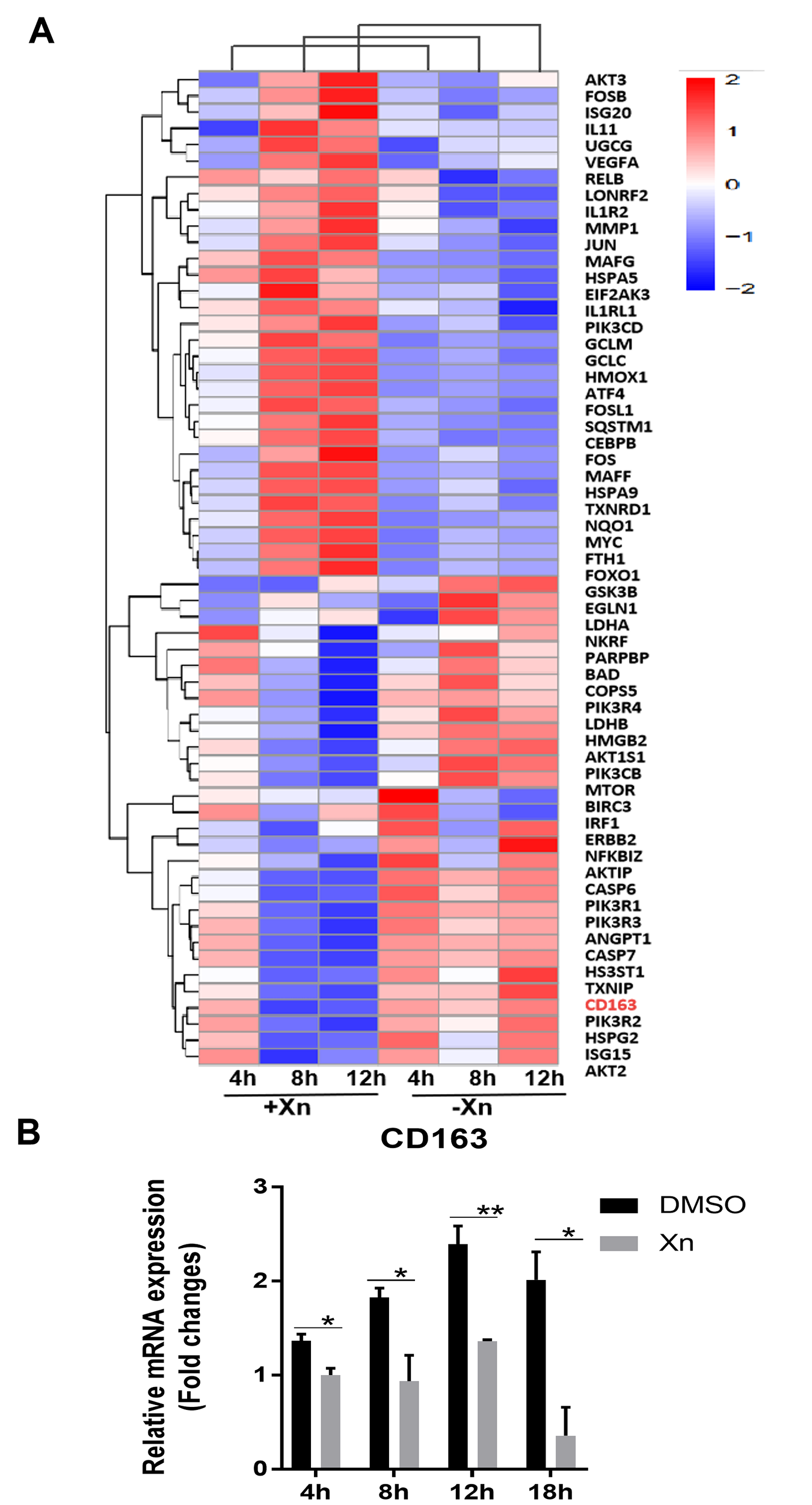

Supplement: Supplementary file 3 — Additional file 3. Xn treatment downregulated the CD163 mRNA levels in Marc-145 cells. (A) Heat map of expressed genes modulated by 10 µM Xn or DMSO treatment in Marc-145 cells. Red and blue correspond to relative up- and downregulation, respectively. (B) Marc-145 cells were treated with 10 µM Xn for 4, 8, 12 and 18 h, followed by qRT-PCR for CD163 mRNA levels. **P < 0.01; *P < 0.05 vs DMSO-treated cells. [file 13567_2019_679_MOESM3_ESM.tif]
